# CircHAS2 activates CCNE2 to promote cell proliferation and sensitizes the response of colorectal cancer to anlotinib

**DOI:** 10.1186/s12943-024-01971-7

**Published:** 2024-03-21

**Authors:** Haosheng Li, Haoran Feng, Tao Zhang, Junwei Wu, Xiaonan Shen, Shuiyu Xu, Lianghui Xu, Shaodong Wang, Yaqi Zhang, Wenqing Jia, Xiaopin Ji, Xi Cheng, Ren Zhao

**Affiliations:** 1 Department of General Surgery, Ruijin Hospital, Shanghai Jiao Tong University School of Medicine, Shanghai, China; 2 Shanghai Institute of Digestive Surgery, Ruijin Hospital, Shanghai Jiao Tong University School of Medicine, Shanghai, China; 3 Department of Oncology, Ruijin Hospital, Shanghai Jiao Tong University School of Medicine, Shanghai, China; 4 Department of Gastroenterology, Ruijin Hospital, Shanghai Jiao Tong University School of Medicine, Shanghai, China

**Keywords:** Colorectal cancer, circRNAs, Anlotinib, Biomarker, Translation

## Abstract

**Background:**

Tyrosine kinase inhibitors (TKIs) are crucial in the targeted treatment of advanced colorectal cancer (CRC). Anlotinib, a multi-target TKI, has previously been demonstrated to offer therapeutic benefits in previous studies. Circular RNAs (circRNAs) have been implicated in CRC progression and their unique structural stability serves as promising biomarkers. The detailed molecular mechanisms and specific biomarkers related to circRNAs in the era of targeted therapies, however, remain obscure.

**Methods:**

The whole transcriptome RNA sequencing and function experiments were conducted to identify candidate anlotinib-regulated circRNAs, whose mechanism was confirmed by molecular biology experiments. CircHAS2 was profiled in a library of patient-derived CRC organoids (*n* = 22) and patient-derived CRC tumors in mice. Furthermore, a prospective phase II clinical study of 14 advanced CRC patients with anlotinib-based therapy was commenced to verify drug sensitivity (ClinicalTrials.gov identifier: NCT05262335).

**Results:**

Anlotinib inhibits tumor growth in vitro and in vivo by downregulating circHAS2. CircHAS2 modulates CCNE2 activation by acting as a sponge for miR-1244, and binding to USP10 to facilitate p53 nuclear export as well as degradation. In parallel, circHAS2 serves as a potent biomarker predictive of anlotinib sensitivity, both in patient-derived organoids and xenograft models. Moreover, the efficacy of anlotinib inclusion into the treatment regimen yields meaningful clinical responses in patients with high levels of circHAS2. Our findings offer a promising targeted strategy for approximately 52.9% of advanced CRC patients who have high circHAS2 levels.

**Conclusions:**

CircHAS2 promotes cell proliferation via the miR-1244/CCNE2 and USP10/p53/CCNE2 bidirectional axes. Patient-derived organoids and xenograft models are employed to validate the sensitivity to anlotinib. Furthermore, our preliminary Phase II clinical study, involving advanced CRC patients treated with anlotinib, confirmed circHAS2 as a potential sensitivity marker.

**Supplementary Information:**

The online version contains supplementary material available at 10.1186/s12943-024-01971-7.

## Introduction

Recent data from the American Cancer Society (2022) indicate that colorectal cancer (CRC) is the second leading cause of cancer-related deaths worldwide [1]. Although the annual CRC mortality rate has been decreasing, this trend masks an increase in mortality among younger individuals [2]. Surgical resection, adjuvant chemotherapy, molecular targeted therapy, and neoadjuvant therapy are currently the primary clinical treatments for CRC [3]. Prospective studies have demonstrated that combining chemotherapy with targeted therapies has significant potential in treating advanced CRC, presenting novel strategic avenues [4]. Targeted drugs have emerged as an effective personalized precision treatment for CRC, and a better understanding of their mechanisms is critical for patient care.

Receptor tyrosine kinases (RTK), which are transmembrane glycoproteins, play an important role in angiogenesis, and Tyrosine kinase inhibitors (TKIs) have been used successfully in the treatment of various solid tumors [5, 6]. Anlotinib, a novel orally administered small-molecule TKI, suppresses angiogenesis and tumor cell proliferation in multiple solid tumors by targeting VEGFR1/2/3, c-Kit, PDGFR-α, FGFR1, FGFR2, and FGFR3 [7]. Anlotinib has a broad range of anti-cancer effects compared to other anti-angiogenesis drugs [8]. Ongoing clinical trials are investigating its efficacy in gastrointestinal tumors and focusing further on its potential in adjuvant treatment [9, 10].

Noncoding genes comprise over 98% of the human genome, with circular RNAs (circRNAs) playing an important role in cancer development and having the potential as biomarkers for cancer diagnosis, prognosis, and treatment [11–13]. Recent findings indicate that circRNAs are expressed differently in CRC patients’ tumor tissues and blood than in healthy controls [14]. In addition, circRNAs are thought to be potential tissue or liquid biopsy biomarkers for CRC diagnosis, susceptibility to targeted drugs, and resistance to antineoplastic drugs with high in vivo stability [15]. circRNAs may function as transcriptional regulators, miRNA sponges, or protein binders in cancer progression [16–18]. Identifying drug-regulated circRNAs has significant translational implications for clinical diagnosis and treatment.

The decision to use targeted drugs is primarily based on histopathological characteristics and genotyping. However, only a small fraction of patients benefit from these approaches [19]. More refined biomarkers are required in such cases, particularly those that indicate the unique sensitivity to a specific targeted drug [20]. Next-generation sequencing and high-throughput technologies have significantly facilitated the discovery of biomarkers and increased our understanding of the potential mechanisms of targeted drugs [21–23]. Because of its short half-life and significant variation in expression with physiological conditions, mRNA is not an ideal predictive factor. In contrast, circRNAs have several advantages over linear mRNA due to structural characteristics and remarkable stability [24].

The present study aims to determine the potential functions and specific mechanisms of circHAS2 in CRC progression and its role in predicting clinical response to anlotinib. Our results showed that CRC patients with high levels of circHAS2 are associated with a poor prognosis. Anlotinib-regulated circHAS2 suppresses CRC cell proliferation in vitro and in vivo through the cell cycle. Mechanistically, circHAS2 acts as a sponge for miR-1244 activating CCNE2-driven cell proliferation, and disrupts the p53 and USP10 binding increasing nuclear export of p53 to ubiquitin degrade and further activation of CCNE2 via p21. Based on our preclinical findings, we commenced a clinical study at Shanghai Ruijin Hospital (ClinicalTrials.gov identifier: NCT05262335) to assess the safety and efficacy of anlotinib inclusion to the initial treatment regimen in patients with advanced CRC. Notably, treatment of patients afflicted with advanced colorectal cancer who have high levels of circHAS2 resulted in substantial clinical remission.

## Materials and methods

### Patient recruitment

A prospective, exploratory, and phase II clinical trial assessed the safety and efficacy of anlotinib combined with chemotherapy as first-line adjuvant therapy for patients with advanced unresectable CRC. The present study was approved by the Ethics Committee of Ruijin Hospital, Shanghai Jiao Tong University School of Medicine, Shanghai, China (Approval No. 2021–223), and it was registered with the Clinical Trial Registry (Registration No. NCT05262335). All patients provided written informed consent before enrollment. Based on the Response Evaluation Criteria in Solid Tumors (RECIST, v1.1) guidelines, inclusion criteria for the study included at least one measurable progressive lesion within the past three months and no prior treatment history [25].

### Specimens and ethics

The present study included 70 patients with a definitive diagnosis of CRC, with both tumor tissue and adjacent non-tumor tissue samples. The Ethics Committee of Ruijin Hospital approved all procedures. All samples were collected for scientific research with informed consent from the patients and their authorized representatives. All specimens were pathologically confirmed and definitively diagnosed using hematoxylin and eosin (H&E) staining before immunohistochemical staining, with no patients receiving radiotherapy, chemotherapy, or biological treatment before surgery. The local infiltration of malignant tumor cells, degree of differentiation, lymph node metastasis, and relevant information on distant metastasis was recorded according to the World Health Organization tumor-node-metastasis (TNM) staging standard.

### Cell culture and treatments

The cell lines SW620, HCT116, SW480, and HT29 were purchased from the Shanghai Institutes for Biological Sciences, Chinese Academy of Sciences. Anlotinib was obtained from MedChemExpress (MCE, USA). circHAS2, circHAS2 siRNA, circHAS2 shRNA, miR-1244 mimic, and miR-1244 inhibitor were synthesized by Bioegene (Bioegene Tech, China). USP10 siRNA was synthesized by Sangon (Sangon Biotech, China). CCNE2, CCNE2 siRNA, CCNE2 shRNA, circHAS2 truncations, Flag-USP10, and HA-Ub, were synthesized by Public Protein/Plasmid Library (PPL, China). Lipofectamine 3000 was used as a transfection reagent for cell transfection, subsequent experiments, and stable cell line generation. All sequences are listed in Supplementary File Table S5.

### Cell counting kit (CCK-8) assay

Cell viability was assessed using the Cell Counting Kit (CCK-8, Meilunbio, China). Briefly, cells in logarithmic growth phase (SW620, SW480, HT29, and HCT116) were seeded at a density of 8,000 cells per well in 96-well cell culture plates. After 24, 48, and 72 h of incubation at a constant temperature, culture medium containing anlotinib at concentrations of 1 µmol/L, 5 µmol/L, 10 µmol/L, 20 µmol/L, 40 µmol/L, and 80 µmol/L was added. Following an additional 24 h of incubation, 100 µL of culture medium with 10% CCK-8 solution was added, and absorbance at 450 nm (OD450) was measured using a microplate reader.

### EdU incorporation assay

To determine cell proliferation rates, we utilized the EdU incorporation assay kit (C0075L, Beyotime). Briefly, cells were treated with anlotinib-containing DMEM or RPMI-1640 medium in six-well plates. After treatment, cells were incubated with the EdU working solution for 2 h, fixed with 4% paraformaldehyde, and subsequently stained with Azide 555 as well as Hoechst 33,342. The percentage of fluorescence-positive cells was then calculated to assess the proliferation rate.

### RNA fluorescence in situ hybridization (FISH) and in situ hybridization (ISH)

FISH and ISH analyses were performed using specific probes for circHAS2 (5’-ACAATGCATCTGTACATAATCCAC-3’) and miR-1244 (5’-TTCATCAACCAAAC

ATACTCTACCAA-3’), designed by SweAMI (Servicebio, China). The fluorescence in situ hybridization kit (Servicebio, China) detected the expression and localization in 70 paired CRC tissue microarrays, SW620 and HCT116 cell lines, and subcutaneous CRC tumors in mice. The circHAS2 expression was assessed through the positive staining intensity (strong = 3, moderate = 2, weak = 1, and negative = 0) and the percentage of positively stained cells (> 76% = 4, 51–75% = 3, 26–50% = 2, 5–25% = 1, and < 5% = 0). Samples were classified into low and high-expression groups based on ISH scoring.

### Dual-luciferase reporter assay

Fluorescent luciferase reporter vectors, containing wild-type and mutant sequences matching the 3’ UTR binding sites of circHAS2 and CCNE2, were synthesized by Public Protein/Plasmid Library, China (PPL, China), and cloned into the pmirGLO reporter plasmid. These reporter plasmids were transfected into SW620 and HCT116 cells. The Dual-Luciferase Reporter Assay Kit (Beyotime, China) was employed to measure luciferase activity, with RLU (relative light units) determined after adding luciferase assay reagents and normalized to Renilla luciferase activity. This comparison indicated the degree of activation of the target reporter genes.

### Immunoprecipitation (IP) assay and RNA immunoprecipitation (RIP)

Immunoprecipitation was conducted using the Pierce Co-Immunoprecipitation (Co-IP) kit (Thermo Fisher, USA) according to the manufacturer’s protocol. Antibodies against USP10 and p53 were added to cell lysates, and target proteins were captured on protein A/G-sepharose beads after forming immune complexes. The eluted antigens were subsequently analyzed by sodium dodecyl sulfate-polyacrylamide gel electrophoresis (SDS-PAGE) followed by Western blotting. For the RIP assay, the RNA-Binding Protein Immunoprecipitation kit (Geneseed, China) was employed. Lysates from SW620 and HCT116 cells were incubated with RIP buffer containing magnetic beads conjugated with antibodies against Flag and USP10. Normal rabbit IgG served as a negative control. In summary, protein A/G beads were pre-treated and coupled with antibodies, followed by incubation with cell lysate supernatant at 4 °C overnight. After washing the bead complexes, RNA was extracted and purified, while proteins were concurrently isolated from the same magnetic bead complexes in the samples. Western Blotting and Quantitative Reverse Transcription Polymerase Chain Reaction (qRT-PCR) were employed to examine enriched target proteins and RNA fragments.

### RNA pull-down assay and mass spectrometry analysis

CircHAS2-associated RNA-binding proteins (RBPs) were identified by circRNA pull-down assays using the MS2 bacteriophage coat protein (MS2-CP) [26, 27]. In essence, two overexpression vectors, one carrying circHAS2-MS2 and the other bearing MS2-CP-Flag, were constructed and labeled with a green fluorescent protein (GFP) and red fluorescent protein (m-Cherry), respectively (Geneseed, China). Both vectors were co-transfected into SW620 cells to induce MS2-CP expression. The Flag-MS2-CP-MS2-circHAS2 complex was pulled down by the anti-Flag antibody after specific binding of MS2-tagged circHAS2 and MS2-CP. Subsequently, the captured products were identified using RT-qPCR or WB. Finally, mass spectrometry (Thermo Scientific Q Exactive) examined the pull-down products and their controls.

### Xenograft tumor model

SW620, HCT116, vector, and circHAS2 overexpression (circHAS2 oe) cells were cultured until the logarithmic growth phase for cell counting. About five weeks old, male nude mice (*n* = 5) were subcutaneously inoculated on the right flank with 100 µL of cell suspension containing 5 × 10^6^ cells. Tumor formation was observed after one week based on caliper measurements using the modified ellipsoidal formula: Tumor volume = 1⁄2 length × width^2^. The mice were administered anlotinib (3 mg/kg, orally, every two days) based on tumorigenicity, and tumor volume was measured before each dose. Following treatment, the xenograft tumors were excised under anesthesia, weighed, and preserved in a formalin solution for subsequent analysis. The survival time of the mice was monitored thereafter.

### Patient-derived organoids (PDO) model

After undergoing laparoscopic surgery at Ruijin Hospital, all patients were pathologically diagnosed with CRC. CRC samples were collected with informed consent and approved by the Medical Ethics Committee of Ruijin Hospital. The surgical samples were washed with PBS containing dual antibiotics and finely minced. The tissue fragments were digested at 37 ˚C for 1 h with 0.5 mg/mL Type IV collagenase (Sigma, USA). The cells were embedded in Matrigel (356,231, Corning, USA) and covered with human intestinal organoid growth medium (WM-H-03, OuMel, China) after filtering the digested tissue suspension to remove residual tissue. In the present study, 22 PDO samples were used, which were divided into two groups based on the high or low expression of circHAS2. A luciferase or enzyme-linked immunosorbent assay system was used to detect cell viability after drug treatment.

### Patient-derived tumor xenograft (PDX) models

In this study, tumor tissues from colon cancer patients were collected in compliance with the Medical Ethics Committee of Ruijin Hospital to establish PDX models. Tumor samples were obtained, finely cut into fragments, and implanted subcutaneously in the anterior flank of anesthetized 6-week-old NSG nude female mice within 3 h. Mice were housed in pathogen-free cages and acclimatized for three days in approved facilities with free access to water and food and controlled environmental conditions. Animal experiments were conducted in accordance with institutional guidelines and principles of animal research, providing daily care. When tumor volume reached 100 mm³, it was further transplanted to NSG mice to form second-generation xenograft tumors. After three passages, once the tumor genetics stabilized, the mice were randomly divided into two groups (*n* = 5). Tumor formation was observed after one week using caliper measurements and the modified ellipsoidal formula: Tumor volume = 1/2 length × width². The mice received anlotinib (3 mg/kg, orally, every two days) based on tumorigenicity, with tumor volume measured before each dose. Post-treatment, the xenograft tumors were excised under anesthesia, weighed, and preserved in formalin for subsequent analysis. The survival time of the mice was also monitored. Anlotinib administration commenced when tumor volume approximated 200 mm³. After seven treatments, the xenograft tumor was removed for survival curve analysis. All procedures and protocols were approved by the Animal Care and Use Committee of Ruijin Hospital.

### Western blotting and immunohistochemistry

Proteins were extracted from cells with lysis buffer and separated on 7.5%, 10%, and 12.5% SDS-PAGE gels before being transferred to a membrane. The membrane was then incubated with the specific primary and secondary antibodies, as presented in Supplementary File Table S6. GAPDH and β-tubulin were used as controls. Immunohistochemical (IHC) staining was performed through a previously described method [28], using antibodies for CCNE2 (Abclonal, A7032), CDK2 (Abclonal, A0294), p53 (Abclonal, A0263), p21 (Abclonal, A19094), and USP10 (Abclonal, A4454). The nuclei were stained with DAPI, and IHC scores were calculated using the previous ISH scores method.

### Statistical analysis

The data were analyzed using GraphPad Prism (version 9.4.1, San Diego, CA, USA). Statistical differences in qPCR, gene expression, clone formation, and EdU experiments were assessed using two-sided Student’s t-test. Cell viability was determined by CCK-8 assay, employing two-way analysis of variance. One-way analysis of variance was utilized for multiple-group comparisons. All results are presented as the mean ± standard deviation (SD) from three independent experiments. Clinical and biological variables between patient groups were evaluated using the chi-square test. Survival analysis was conducted using the Log-rank method. Symbols (*), (**), and (***) denote *p* < 0.05, *p* < 0.01, and *p* < 0.001, respectively.

## Results

### The efficacy of anlotinib in CRC

Anlotinib, a novel multi-target TKI approved in China, exhibits significant anti-tumor activity against VEGFR. It has been demonstrated to inhibit the PI3K/AKT, MAPK/ERK, and RAF/MRK pathways, showing potential in treating various cancers [8]. Our preliminary in vitro findings suggest that anlotinib may exert direct cytotoxic effects on CRC cell lines SW620, HT29, SW480, and HCT116, thus assessing its efficacy. The results consistently show that anlotinib inhibits CRC cell line proliferation in a dose- dependent and time-dependent manner (Fig. S1A, B). Furthermore, the EdU incorporation and colony formation assays confirmed significant inhibition of SW620 and HCT116 proliferation by anlotinib (Fig. S1C, D). Flow cytometry analysis of cell cycle progression in SW620 and HCT116 cells indicated a decrease in S phase cells and an increase in G0/G1 phase cells under anlotinib treatment, hindering cell entry into the S phase (Fig. S1E). To explore the specific mechanism of anlotinib in colorectal cancer, mRNA sequencing was performed in the SW620 cell line. This sequencing revealed alterations in various tumor-related signaling pathways, including transcriptional misregulation in cancer, cell cycle, and the p53 signaling pathway (Fig. S1F). Further examination of anlotinib’s regulatory effect on the CRC cell cycle, using Western blot analysis of SW620 and HCT116 cells for CCNE1, CCNE2, CDK2, CCNA2, CDK1, and Histone H3, showed reduced protein levels following anlotinib treatment, especially at high concentrations (Fig. S1G). In vivo, xenograft models using CRC cell lines SW620 and HCT116 were established to validate anlotinib’s inhibitory effects. Anlotinib was administered at one-day intervals, and subcutaneous tumors were collected post-treatment. Treatments consistently led to reductions in tumor volume and weight (Fig. S2A-C), without significant differences in body weight, indicating its well tolerability (Fig. S2D). Anlotinib treatment also markedly increased survival in treated mice (Fig. S2E). The proliferation marker Ki-67 and microvascular density marker CD31 were reduced, but no difference was observed in the apoptosis marker TUNEL (Fig. S2F). In summary, Anlotinib exhibits promising anti-tumor effects on CRC cells both in vitro and in vivo.

### Anlotinib-regulated circHAS2 highly expressed in CRC

**Fig. 1:**
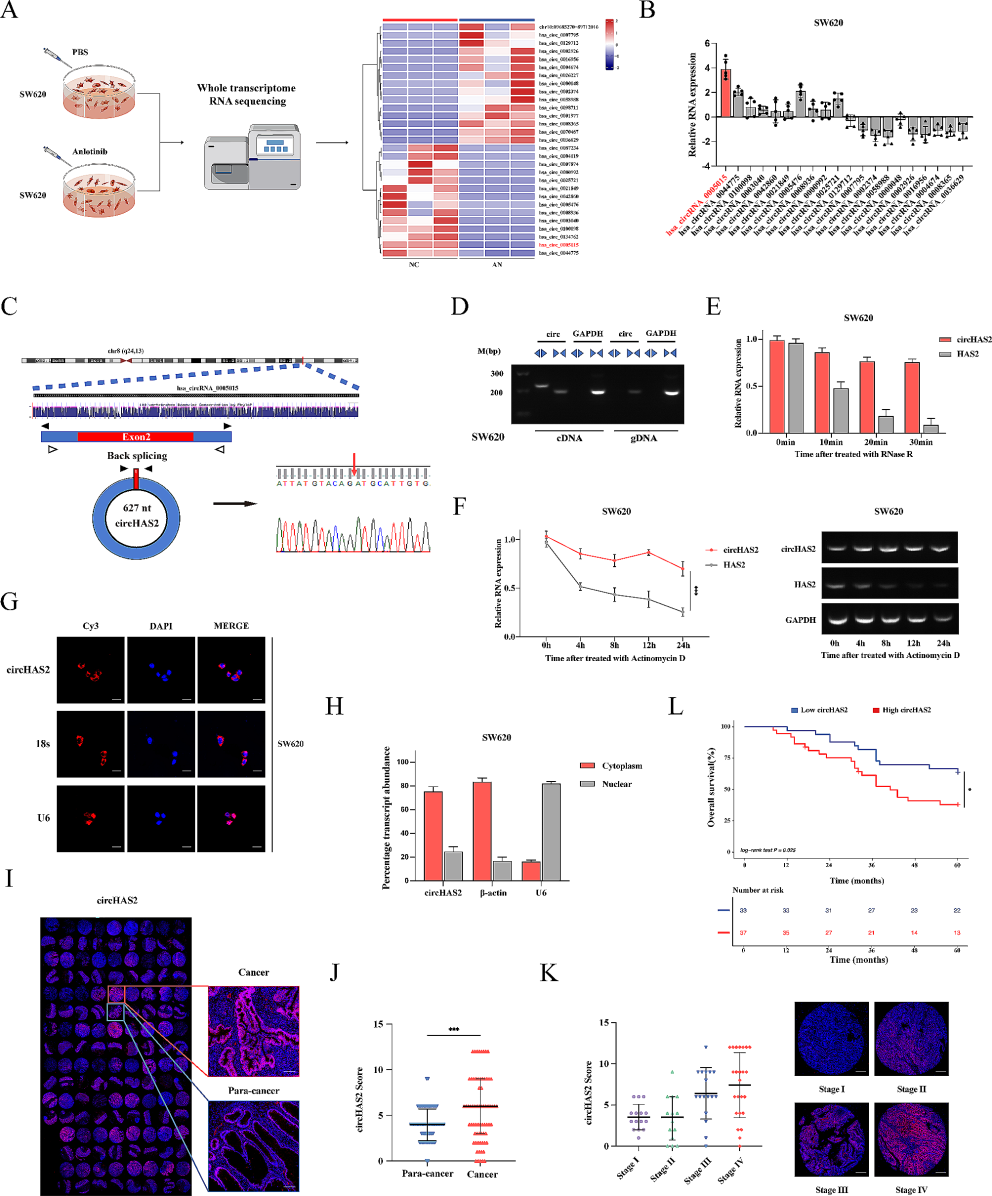
Characteristic of circHAS2 and identification as a biomarker of CRC. **A** Schematic diagram and heatmap depicting whole-transcriptome RNA sequencing results from three independent replicates of SW620 cells cultured with either vehicle control or anlotinib (10 µM) for 24 h. **B** Relative expression of top 10 most upregulated and downregulated circRNAs in 5 CRC tumor tissues and paired normal tissues. **C** Schematic diagram illustrating the formation of circHAS2 from host gene and the back-splice junction site verified by Sanger sequencing. **D** PCR and agarose gel electrophoresis analysis in SW620 cells verified that the divergent primers for circHAS2 could be amplified from cDNA but not gDNA. **E** Relative expression of circHAS2 and HAS2 mRNA after treatment with RNase R in SW620 cells. **F** RNA abundance of circHAS2 and HAS2 mRNA after treatment with Actinomycin D in PCR and agarose gel electrophoresis analysis in SW620 cells. **G** Representative images of the FISH assay in SW620 cells revealed circHAS2 predominantly located in the cytoplasm with the target probe labeled with Cy3 and nuclei stained with DAPI (scale bar: 20 μm). **H** Nuclear and cytoplasm fractionation assays in SW620 cells showed the subcellular location of circHAS2. **I** The expression levels of circHAS2 in CRC tissues and paired normal tissues of microarrays were verified by FISH (scale bar: 100 μm). **J** The expression levels of circHAS2 in CRC tissues and paired normal tissues of microarrays were confirmed by IHC scores. **K** The expression of circHAS2 in different TNM stages was detected by FISH (scale bar: 400 μm). **L** Overall survival curves of 70 CRC patients based on circHAS2 expression levels in our research center. Statistical significance in two-group experiments was evaluated using a two-sided Student’s t-test, and survival analysis was conducted using the Log-rank method. Data are represented as mean ± SD; *, *P* < 0.05; **, *P* < 0.01; ***, *P* < 0.001; ns, no significance

Noncoding RNAs (ncRNAs) play pivotal roles in cancer regulation, yet their involvement in drug mechanisms remains elusive. To address this gap, we conducted whole transcriptome RNA sequencing in SW620 cells treated with anlotinib (Fig. 1A). Our analysis revealed 113 differentially expressed lncRNAs and 29 differentially expressed circRNAs, all exhibiting a fold-change > 2 and a *P*-value < 0.05 (Fig. S3A, H). Subsequently, qRT-PCR was performed to assess top 10 up-regulated and down-regulated ncRNAs in five cases of CRC tumors and matched normal tissues, confirming that hsa_circRNA_0005015 exhibited the most significant up-regulation in tumors and the most pronounced down-regulation under anlotinib treatment in SW620 and HCT116 cells (Fig. 1B and S3B). Based on data from the UCSC Genome Browser (https://genome.ucsc.edu/index.html), hsa_circRNA_0005015 originates from the back-splicing of exon 2 of the HAS2 gene located on chromosome 8, with a length of 627nt. Sanger sequencing verified the splicing site, leading us to designate hsa_circRNA_0005015 as circHAS2 (Fig. 1C). Divergent primers for circHAS2 amplified cDNA but not gDNA in PCR analysis, with amplification products analyzed in SW620 and HCT116 cells by agarose gel electrophoresis. GAPDH served as a negative control (Fig. 1D and S3C). Further investigation involving RNase R and actinomycin D treatments revealed that circHAS2 exhibited greater stability in cells compared to linear HAS2 mRNA in SW620 and HCT116 cells (Fig. 1E, F and S3D, E). Moreover, nuclear-cytoplasmic fractionation and the FISH assay demonstrated that circHAS2 predominantly localized in the cytoplasm in SW620 and HCT116 cells (Fig. 1G, H and S3F, G). These findings collectively indicate that circHAS2 is highly expressed and stable in colorectal cancer cell lines, and is regulated by anlotinib.

CircHAS2 expression was also studied in tissue microarrays with 70 CRC patient tumor samples and matched adjacent normal tissues. The results revealed significantly higher circHAS2 levels in tumor tissues compared to adjacent non-tumor tissues (Fig. 1I, J). Subsequently, RT-PCR analysis of 70 tumor samples and their paired normal tissues revealed that circHAS2 was significantly up-regulated in tumors (Fig. S3J). This high expression was closely related to pathological staging and T-stage of patients (Fig. 1K and S3I). Detailed clinical data analysis was conducted to evaluate circHAS2’s clinical and prognostic significance in CRC. We found a significant correlation between circHAS2 expression and pathological stage, T-stage, and lymph node metastasis (Table S1). Cox regression analysis identified circHAS2 expression as an independent prognostic marker for CRC patients (Table S2). ROC curve analysis demonstrated the circHAS2 level could effectively distinguish CRC from non-tumor tissue, with an AUC of 0.759 (Fig. S3K). Kaplan-Meier survival analysis revealed that high circHAS2 expression correlates with poor prognosis in CRC patients (Fig. 1L and S3L, M).

### Promotion of anlotinib-regulated circHAS2 in CRC proliferation

**Fig. 2:**
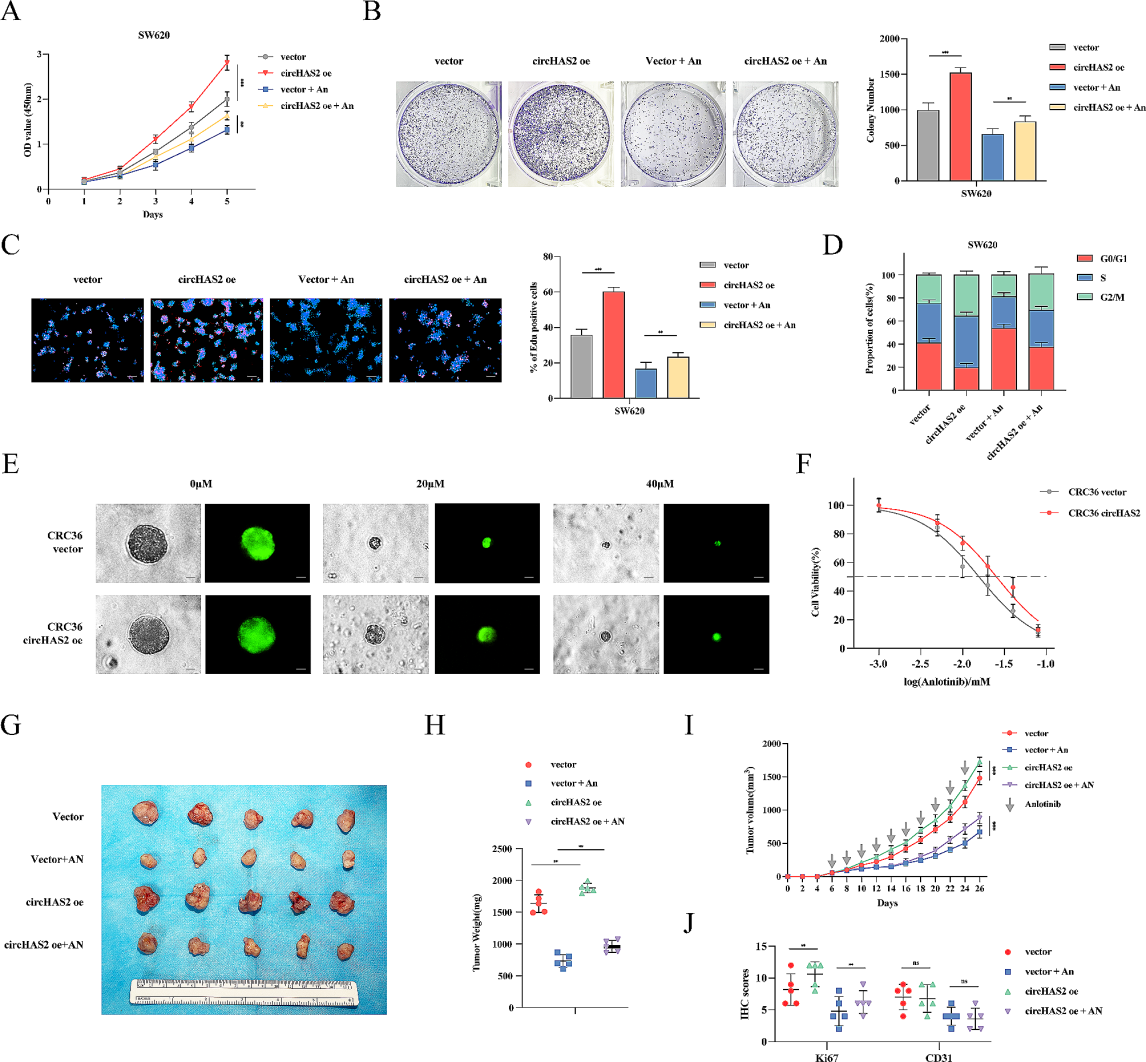
Promotion of Anlotinib-regulated circHAS2 in CRC proliferation in vitro and in vivo. **A** The viabilities of SW620/vector, SW620/circHAS2 oe with the addition of vehicle control or anlotinib were detected by CCK-8 assays in SW620 cells. **B** Quantitative results and representative images of cell proliferation were conducted by colony formation assay with indicated treatment in SW620 cells. **C** Quantitative results and representative images of cell proliferation were evaluated by EdU incorporation with indicated treatment in SW620 cells (scale bar: 50 μm). **D** The percentage of cells in the G1, S, and G2 phases of the whole cell population was determined by flow cytometry with indicated treatment in SW620 cells. **E** Representative images of organoids in the vector- and circHAS2 overexpression-treated groups treated with the indicated concentrations of anlotinib (scale bar: 10 μm). **F** Dose–effect curves of organoids in the vector- and circHAS2 overexpression-treated groups treated with the indicated concentrations of anlotinib. **G** Images of subcutaneous tumors formed by SW620/vector, SW620/circHAS2 oe cells (*n* = 5). The mice were administered either a vehicle control or anlotinib (3 mg/kg, orally, every two days), respectively. **H** Tumor weights were analyzed at the endpoint. **I** Tumor volumes were measured every two days. **J** IHC scores of IHC staining (Ki-67 and CD31) assay of tumors of indicated treatment. Cell viability was determined by CCK-8 assay, employing two-way analysis of variance. Statistical differences in clone formation, EdU experiments, tumor weights, tumor volumes and IHC scores were assessed using two-sided Student’s t-test. Survival analysis was performed using the Log-rank method. Data are represented as mean ± SD; *, *P* < 0.05; **, *P* < 0.01; ***, *P* < 0.001; ns, no significance

Our study aimed to delineate circHAS2’s role in CRC by overexpressing it in SW620 cell lines across four groups: vector, circHAS2 oe, vector with anlotinib, and circHAS2 oe with anlotinib (Fig. S4A). CRC cell proliferation, assessed through CCK-8, colony formation, EdU incorporation, and cell cycle analysis via flow cytometry, showed that high circHAS2 expression significantly accelerated proliferation, while anlotinib attenuated the proliferative promotion in both cell lines (Fig. 2A-D and S4B-F). Employing Patient-derived organoids (PDO) models, we observed that CRC36 vector organoids (IC50 = 15.68 µM) were more sensitive to anlotinib than those with circHAS2 overexpression (IC50 = 25.35 µM), aligning with cell line findings (Fig. 2E, F). In vivo, a xenograft model using SW620 cells confirmed anlotinib’s inhibitory effect on tumor growth, with circHAS2 overexpression countering this effect (Fig. 2G-I). There was no statistically significant difference in body weight among groups (Fig. S4G). Immunohistochemical analysis using anti-Ki67 and CD31 antibodies on xenograft tissues indicated increased Ki67 in the circHAS2 overexpression group, while CD31 levels were unaffected (Fig. 2J and S4H). These results support the notion that circHAS2 promotes CRC tumor growth and is modulated by anlotinib treatment.

### CircHAS2 as a miR-1244 sponge to regulate CCNE2 in CRC

**Fig. 3:**
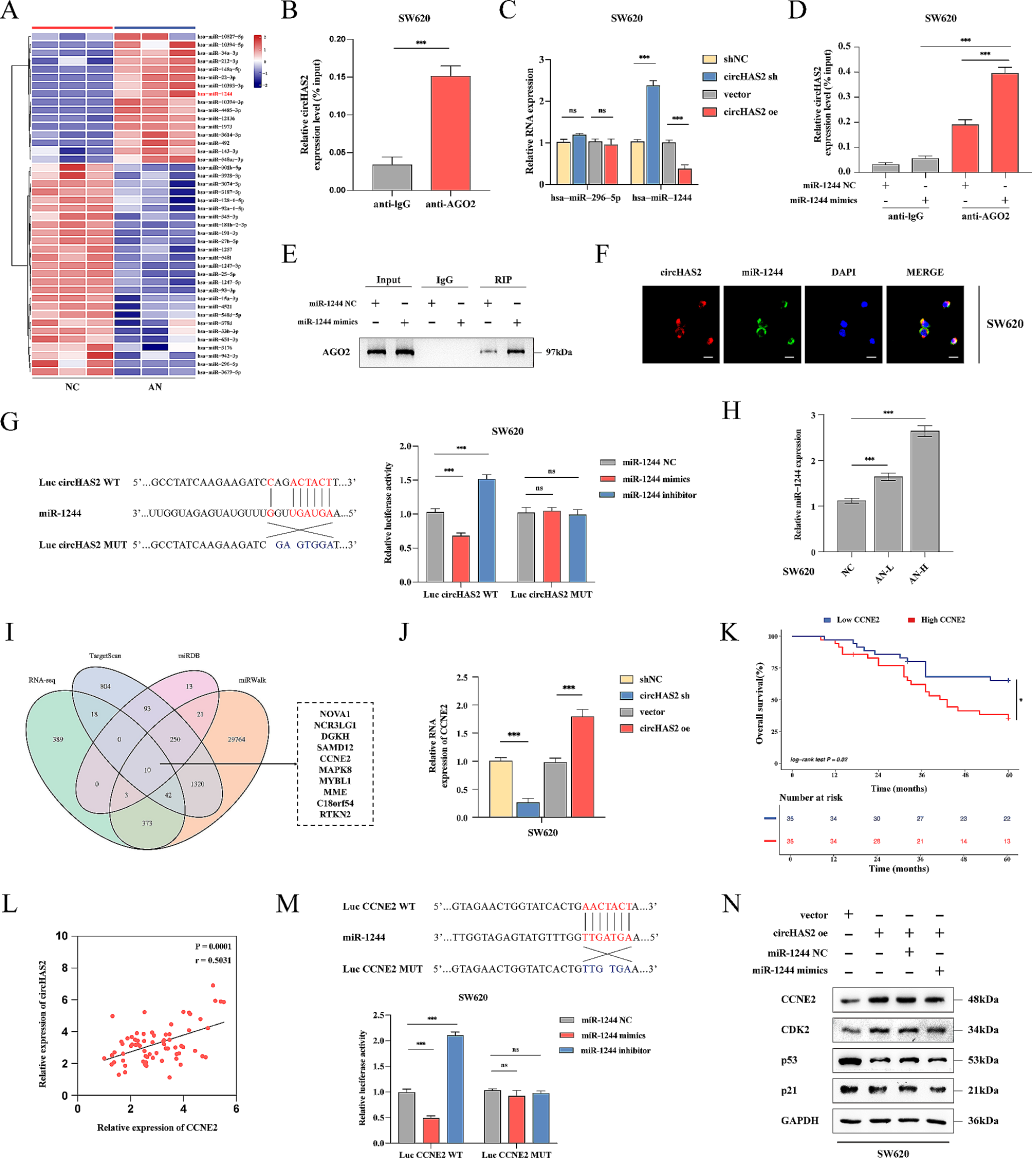
CircHAS2 as a miR-1244 sponge to modulate CCNE2 expression in CRC. **A** Heatmap showing differentially expressed miRNAs in SW620 cells treated by either vehicle control or anlotinib (10 µM) for 24 h. **B** The relative expression of circHAS2 in SW620 cells was analyzed by RIP assay with AGO2 and IgG antibodies. **C** Relative miRNAs expressions were verified after indicated treatment in SW620 cells. **D** The RIP assay was used to detect the relative expression of circHAS2 in SW620 cells with indicated treatment. **E** The RIP assay was used to detect the expression of AGO2 protein in SW620 cells with indicated treatment. **F** Representative images of the FISH assay revealed the colocalization of circHAS2 and miR-1244 in the cytoplasm of SW620 cells with the target probe labeled with Cy3 and nuclei stained with DAPI (scale bar: 20 μm). **G** Luciferase activity of circHAS2 in SW620 cells was verified with co-transfected Luc circHAS2 WT or MUT and miR-1244 mimics or inhibitor. **H** Relative miRNA expressions were confirmed after anlotinib treatment (L: 5 µM, H: 10 µM) in SW620 for 24 h. **I** Prediction of potential downstream target genes for miR-1244 using RNA sequencing, TargetScan, miRDB, and miRWalk. **J** Relative CCNE2 expressions were verified after indicated treatment in SW620 cells. **K** Overall survival curves of 70 CRC patients based on CCNE2 expression levels in our center. **L** The correlation between circHAS2 and CCNE2 was determined by Pearson correlation analysis from our center. **M** Luciferase activity of CCNE2 in SW620 cells was verified with co-transfected Luc CCNE2 WT or MUT and miR-1244 mimics or inhibitor. **N** Representative Western blotting images in SW620 cells with indicated treatments for CCNE2, CDK2, p53, and p21 proteins. GAPDH was used as an internal control. Statistical significance in two-group experiments was evaluated using a two-sided Student’s t-test. One-way analysis of variance was employed for multiple-group comparisons. Survival analysis was performed using the Log-rank method. Data are represented as mean ± SD; *, *P* < 0.05; **, *P* < 0.01; ***, *P* < 0.001; ns, no significance

Most circRNAs in the cytoplasm act as competitive endogenous RNAs (ceRNAs) by sponging for miRNAs [29]. Based on previous research, we hypothesized that circHAS2, predominantly found in the cytoplasm, might exert its effects by competitively inhibiting miRNAs. We used miRNA sequencing in SW620 cells to screen for differentially expressed miRNAs in anlotinib-treated and control groups (Fig. 3A). AGO2, essential for circRNA-miRNA interaction, was found enriched with circHAS2, suggesting circRNA-miRNA binding through AGO2 by RIP assays in SW620 cells (Fig. 3B). Cross-analysis of the sequencing results and the online prediction database CircBank predicted miR-1244 and miR-29a-5p as downstream targets (Fig. S5A). Subsequent experiments confirmed miR-1244 was identified as a circRNA-regulated candidate gene through verification following the knockdown or overexpression of circHAS2 in SW620 cells (Fig. 3C). AGO2 RIP assays then revealed a higher relative enrichment of circHAS2 and AGO2 antibody binding in miR-1244 mimic-transfected cells compared to the miR-1244 NC group (Fig. 3D, E). The co-localization of circHAS2 and miR-1244 was observed in both cell lines (Fig. 3F and S5B). In our 70-patient cohort, miR-1244 was significantly down-regulated in CRC tumors, negatively correlating with circHAS2 and indicating poor prognosis (Fig. S5D-F). Dual-luciferase reporter assays using circHAS2 WT and MUT plasmids in SW620 and HCT116 cells demonstrated that circHAS2 directly bound and regulated miR-1244 (Fig. 3G and S5C). Moreover, miR-1244 was up-regulated under anlotinib treatment in the SW620 cell line (Fig. 3H).

To explore the underlying functions and mechanisms of miR-1244 in CRC, we used TargetScan, miRDB, miRWalk databases, and RNA sequencing cross-analysis to identify ten potential target genes, including NOVA1, NCR3LG1, and DGKH (Fig. 3I). CCNE2 emerged as a co-regulated target, negatively correlating with miR-1244 and positively with circHAS2 (Fig. 3J and S5G, I). Furthermore, using our specimen database center, we identified that CCNE2 was up-regulated in tumors and associated with poor prognoses (Fig. 3K and S5H). CCNE2 expression correlated negatively with miR-1244 expression and positively with circHAS2 expression (Fig. 3L and S5J). Notably, CCNE2 is regulated by multiple genes and pathways and is involved in the CRC cell cycle and proliferation [30]. Based on these findings, we proposed that CCNE2 is a miR-1244 functional target gene. Dual-luciferase reporter assays in SW620 and HCT116 cells revealed miR-1244 binding to the 3’-UTR of CCNE2 (Fig. 3M and S5K). Therefore, we propose that circHAS2 appears to regulate CCNE2 through miR-1244 inhibition, with circHAS2 overexpression activating the CCNE2/CDK2 axis. Nevertheless, transfecting miR-1244 mimics partially reversed these effects, without influencing p53 and p21 levels (Fig. 3N and S5L). Additional potential mechanisms may also contribute to regulating CCNE2 expression.

### Identification of circHAS2 binding to USP10

**Fig. 4:**
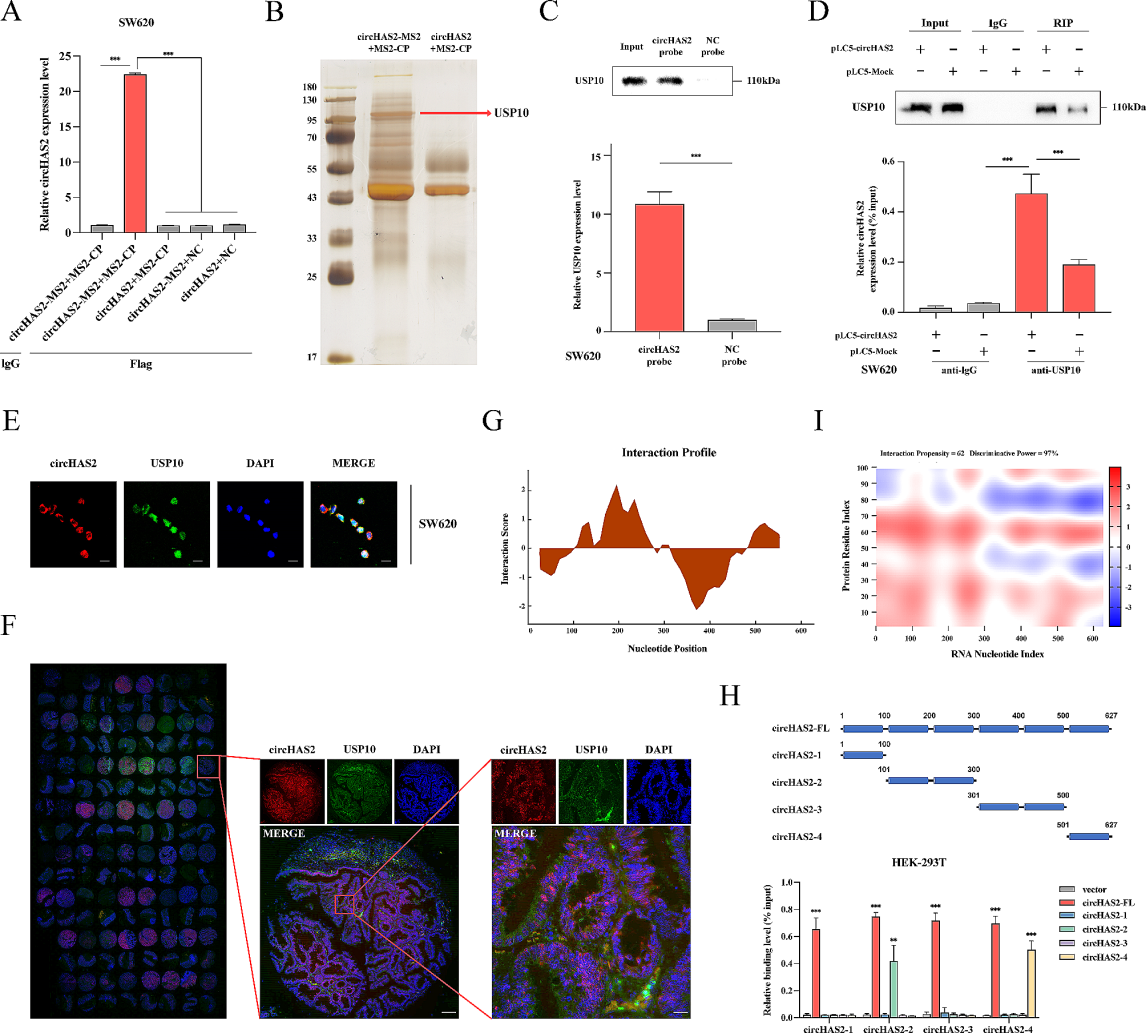
Interaction and co-localization of circHAS2 with USP10. **A** The enrichment of circHAS2-MS2 complex formation was detected with MS2-CP-Flag in SW620 cells. **B** The Silver staining of circHAS2-associated proteins by the biotin-labeled probe. **C** The RNA pull-down confirmed the interaction between circHAS2 and USP10. The relative expression of circHAS2 was determined using the RIP assay with AGO2 and IgG antibodies in SW620 cells. **D** The RIP assays were conducted under the specified treatment conditions using anti-USP10 and anti-IgG antibodies in SW620 cells. **E** Representative images of the FISH assay in SW620 cells revealed the colocalization of circHAS2 and USP10 in the cytoplasm with the target probe labeled with Cy3 and nuclei stained with DAPI (scale bar: 20 μm). **F** The colocalization of circHAS2 and USP10 in CRC tissues and paired normal tissues of microarrays were verified by FISH (scale bar: 400 μm; scale bar: 100 μm). **G** The interaction profile between circHAS2 fragments and USP10 was predicted by the catRAPID database. **H** The interaction between circHAS2 full-length and truncations with USP10 was validated using RIP assays in HEK-293T cells. **I** The heat map illustrated the interaction between circHAS2 and the N-terminal region (1-100 amino acids) of USP10 by the catRAPID database. Statistical significance in two-group experiments was assessed using a two-sided Student’s t-test, and multiple-group comparisons were conducted through one-way analysis of variance. Data are represented as mean ± SD; *, *P* < 0.05; **, *P* < 0.01; ***, *P* < 0.001; ns, no significance

CircRNAs, known for their roles as miRNA sponges and protein interaction platforms, can form functional circRNP complexes and modulate mRNA expression [31]. Using an RNA pull-down assay and silver staining in SW620 cells, we investigated whether circHAS2 fulfilled its functional role by interacting with RNA-binding proteins (Fig. S6A). We generated MS2-CP-MS2-circRNA complexes in SW620 cells through co-transfection with circHAS2-MS2 and MS2-CP vectors (Fig. S6B). Pull-down assays confirmed the specificity of these complexes, showing significant circHAS2 enrichment (Fig. 4A). Subsequent mass spectrometry analysis and silver staining of the differential protein expression profiles between circHAS2-MS2 + MS2-CP and circHAS2 + MS2-CP pull-down experiments identified 110 kDa ubiquitin specific peptidase 10 (USP10) as a likely circHAS2 associate, suggesting its presence in high quantities in circHAS2 protein complexes (Fig. 4B and Table S3). Direct interaction between USP10 and circHAS2 was validated by RNA pull-down and RIP assays of SW620 cells, with a noted increase in USP10 enrichment correlating with circHAS2 expression levels (Fig. 4C, D). FISH analysis demonstrated circHAS2 and USP10 colocalization in the cytoplasm in both cell lines (Fig. 4E and S6C). CircHAS2 and USP10 co-localized at the tissue level in tissue microarrays containing 70 CRC tumor samples and matched adjacent normal tissues (Fig. 4F). The binding affinity between USP10 and circHAS2 was analyzed using the catRAPID database, revealing strongest interactions at specific regions of circHAS2 and USP10. We identified the strongest binding between circHAS2 at positions 100–300 nt and 500–627 nt, and USP10 at positions 1–100 aa (Interaction Propensity = 62, Discriminative Power = 97%) (Fig. 4G, I). Focused on these findings, we investigated the interaction regions between circHAS2 and USP10 using various truncated circHAS2 forms in HEK-293T cells. RIP analysis indicated interactions predominantly between circHAS2-2 and circHAS2-4 with USP10 (Fig. 4H). However, altering circHAS2 expression did not impact USP10 levels at mRNA and protein stages (Fig. S6D-E), and using miR-1244 mimics and inhibitors showed no regulation of USP10 mRNA expression levels (Fig. S6F). Additionally, modulating USP10 expression did not alter circHAS2 and miR-1244 levels, indicating a complex interaction mechanism warranting further study (Fig. S6G-H).

### Disruption of USP10-p53 interaction by circHAS2

**Fig. 5:**
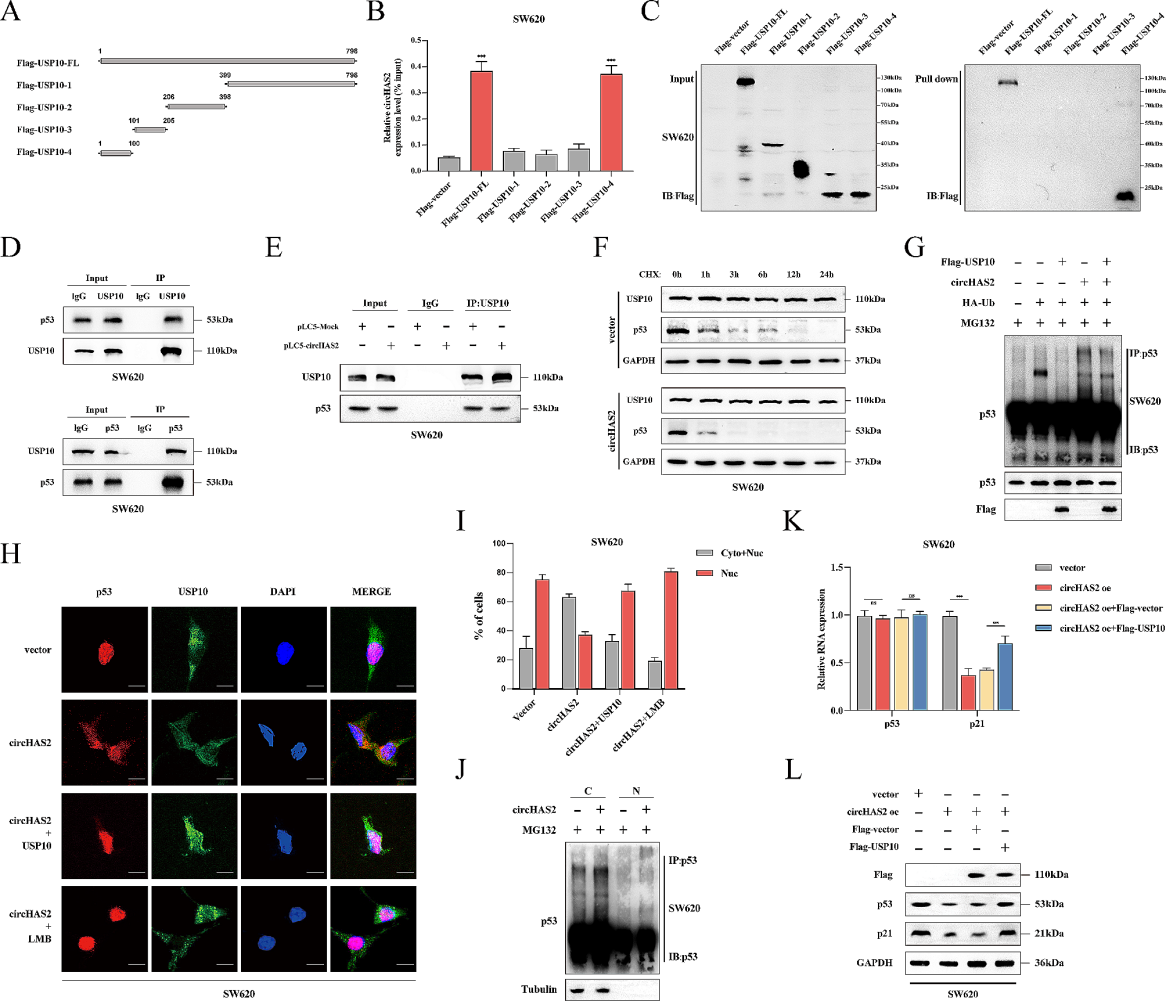
CircHAS2 interaction with USP10 to enhance p53 Ubiquitination. **A** Schematic diagram of USP10 full-length and truncations. **B** The RIP assay was performed in SW620 cells using anti-Flag antibodies to assess the expression levels of circHAS2 following transfection with USP10 full-length and truncations. **C** The RNA pull-down assays were performed using biotin-labeled circHAS2 probes to capture interacting proteins and detect the expression of USP10 full-length and truncations by representative Western blotting images in SW620 cells. **D** The direct interaction between USP10 and p53 was analyzed by Co-IP assays with USP10 and p53 antibodies in SW620 cells. **E** The Co-IP assays were conducted under the specified treatment conditions using USP10 and p53 antibodies in SW620 cells. **F** After indicated treatments were treated with various durations of cycloheximide (0.1 mg/ml) in SW620 cells, and the expression of p53 was detected by Western blotting. GAPDH was employed as an internal control. **G** CircHAS2 modulated the levels of p53 ubiquitination through the interaction with USP10. SW620 cells were transfected with the indicated constructs and subjected to MG132 (50 mM) for 4 h before harvest to evaluate the ubiquitination levels of p53. **H** Representative images of the FISH assay revealed the localization of p53 after indicated treatments with the target probe labeled with Cy3 and nuclei stained with DAPI in SW620 cells (scale bar: 20 μm). **I** Quantification of cells with different p53 subcellular localization after indicated treatments in SW620 cells. Nuc, Nucleus only; Cyto + Nuc, both cytoplasm and nucleus. **J** SW620 cells were transfected with the indicated constructs and subjected to MG132 treatment to evaluate the ubiquitination levels of cytoplasmic or nuclear p53. **K** Relative p53 and p21 expressions were verified after indicated treatments in SW620 cells. **L** Representative Western blotting images with indicated treatments in SW620 cells for Flag, p53, and p21 proteins. GAPDH was used as an internal control. Statistical significance in two-group experiments was assessed using a two-sided Student’s t-test. Data are represented as mean ± SD; *, *P* < 0.05; **, *P* < 0.01; ***, *P* < 0.001; ns, no significance

**Fig. 6:**
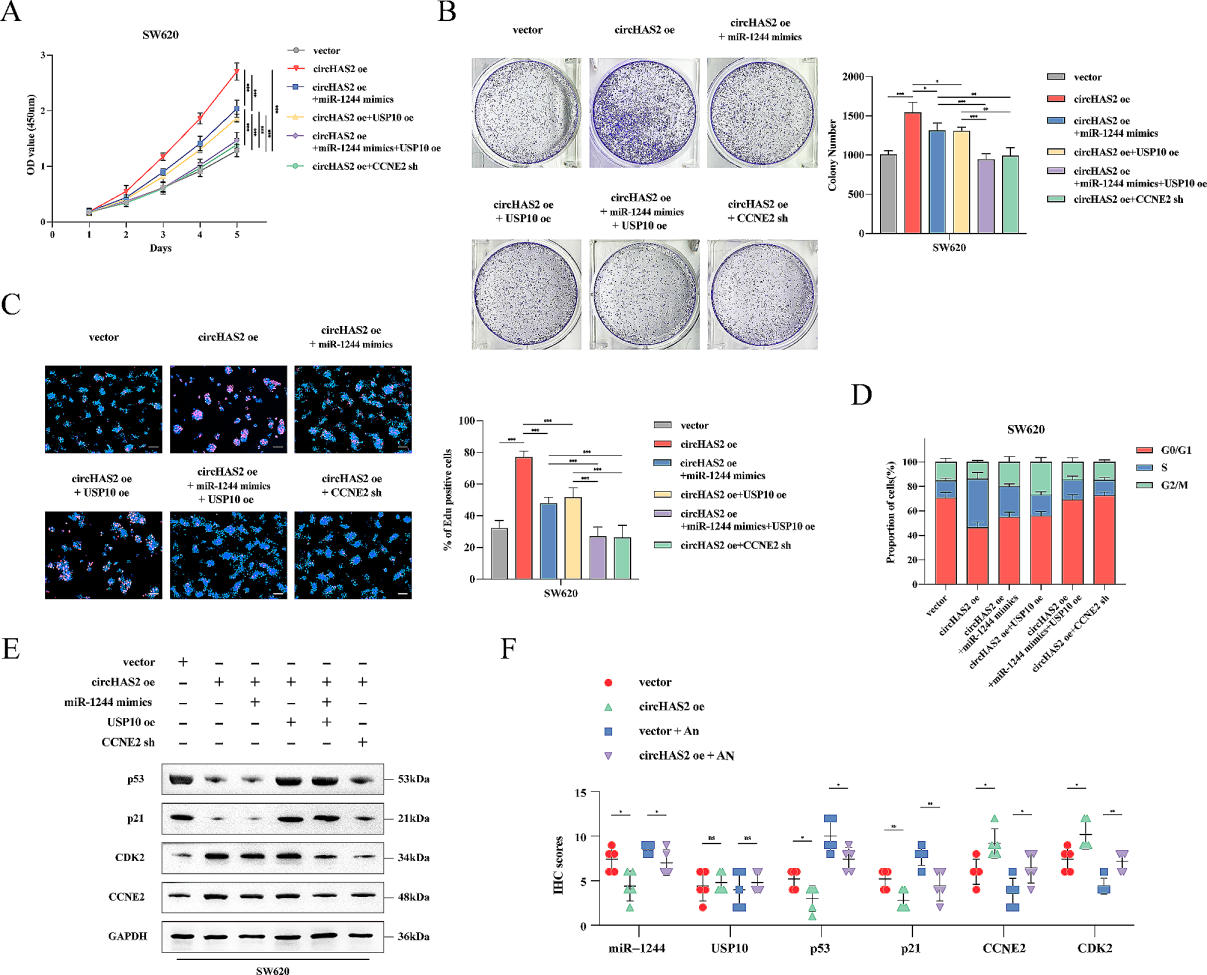
CircHAS2 activating bidirectional downstream pathways to promote CRC progression. **A** The viabilities of SW620 cells following indicated treatments were detected by CCK-8 assays. **B** Quantitative results and representative images of cell proliferation were conducted by colony formation assay in SW620 cells following indicated treatments. **C** Quantitative results and representative images of cell proliferation were evaluated by EdU incorporation in SW620 cells following indicated treatments (scale bar: 50 μm). **D** The percentage of cells in the G1, S, and G2 phases of the entire cell population was determined by flow cytometry following indicated treatments in SW620 cells. **E** Representative Western blotting images with indicated treatments for p53, p21, CDK2, and CCNE2 proteins in SW620 cells. GAPDH was used as an internal control. **F** IHC scores of ISH staining (miR-1244) and IHC staining (USP10, p53, p21, CCNE2, and CDK2) assay of tumors of indicated treatments. Cell viability was determined by CCK-8 assay, employing two-way analysis of variance. Statistical differences in clone formation, EdU experiments, and IHC scores were assessed using two-sided Student’s t-test. Data are represented as mean ± SD; *, *P* < 0.05; **, *P* < 0.01; ***, *P* < 0.001; ns, no significance

To investigate the effect of circHAS2 on the USP10, we constructed Flag-tagged full-length and truncated proteins of USP10’s functional domains (Fig. 5A). RIP assays and RNA pull-down assays in SW620 cells identified the N-terminal region (1–100 aa) of USP10 as critical for circHAS2 binding (Fig. 5B-C), confirming their direct interaction. These findings supported the direct binding between circHAS2 and the deubiquitinating enzyme USP10. Previous studies have reported that USP10 can bind to p53 and stabilize its expression in HCT116 cells [32]. We performed Co-IP assays to confirm the presence of this interaction, which revealed that USP10 and p53 could immunoprecipitate each other (Fig. 5D). This led us to hypothesize that circHAS2 might disrupt USP10-p53 interactions. RIP assays in SW620 cells with circHAS2 overexpression supported this, showing increased binding between overexpressed circHAS2 and USP10 diminished p53 enrichment (Fig. 5E). Furthermore, ubiquitination-related experiments in SW620 and HCT116 demonstrated that circHAS2 overexpression increased p53 protein ubiquitination, resulting in reduced expression, which could be reversed by USP10 (Fig. 5F, G and S6I, J). P53 is readily visible in the cell nucleus, where it is primarily active, and USP10 modulates the nuclear export mechanism of p53, causing it to be ubiquitinated in the cytoplasm [33]. We observed that circHAS2 increased the p53 nuclear export (Fig. 5H), confirmed by cell fractionation experiments of SW620 cells, suggested that circHAS2 induces p53 ubiquitination and nuclear export, counteracted by USP10 co-expression or LMB treatment (Fig. 5I). In addition, circHAS2 overexpression increased p53 ubiquitination in the cytoplasm but not in the nucleus (Fig. 5J). RT-qPCR results indicated that circHAS2 overexpression that altered USP10 without affecting p53 mRNA levels, but downregulated p21 mRNA (Fig. 5K). CircHAS2 reduced p53 and p21 protein levels in CRC cells, partially reversed by USP10 in both cell lines (Fig. 5L and S6K). Overall, our findings in Figs. 5 and 6 underscored circHAS2’s pivotal role in disrupting p53-USP10 binding and influencing p53 nuclear localization.

### CircHAS2 activating bidirectional downstream pathways to enhance CRC growth

To elucidate circHAS2’s specific functions in CRC, we established six experimental groups: control, circHAS2 oe, circHAS2 oe + miR-1244 mimics, circHAS2 oe + USP10 oe, circHAS2 oe + USP10 oe + miR-1244 mimics, and circHAS2 oe + CCNE2 sh in both cell lines. CCK-8, colony formation, EdU assays, and flow cytometry analyses indicated enhanced proliferation in circHAS2-oe groups, mitigated by miR-1244 or USP10, and fully reversed by their combination or CCNE2-knockdown (Fig. 6A-D and S7A-E). Furthermore, USP10 restoration significantly increased p53 and p21 expression, while miR-1244 and USP10 affected CDK2 and CCNE2 expressions (Fig. 6E and S8A). At the tissue level, we performed ISH with miR-1244 probes and immunohistochemical staining with antibodies against USP10, p53, p21, CCNE2, and CDK2. Post-anlotinib treatment showed that miR-1244 expression levels increased, p53 and p21 expressions were up-regulated, while CCNE2 and CDK2 were down-regulated, indicating tumor-suppressive functions (Fig. 6F and S8B). These results suggest that anlotinib-regulated circHAS2 downregulation inhibits CRC cell growth through ceRNA mechanisms and p53 disruption.

### CircHAS2 as a predictive biomarker for anlotinib sensitivity

**Fig. 7:**
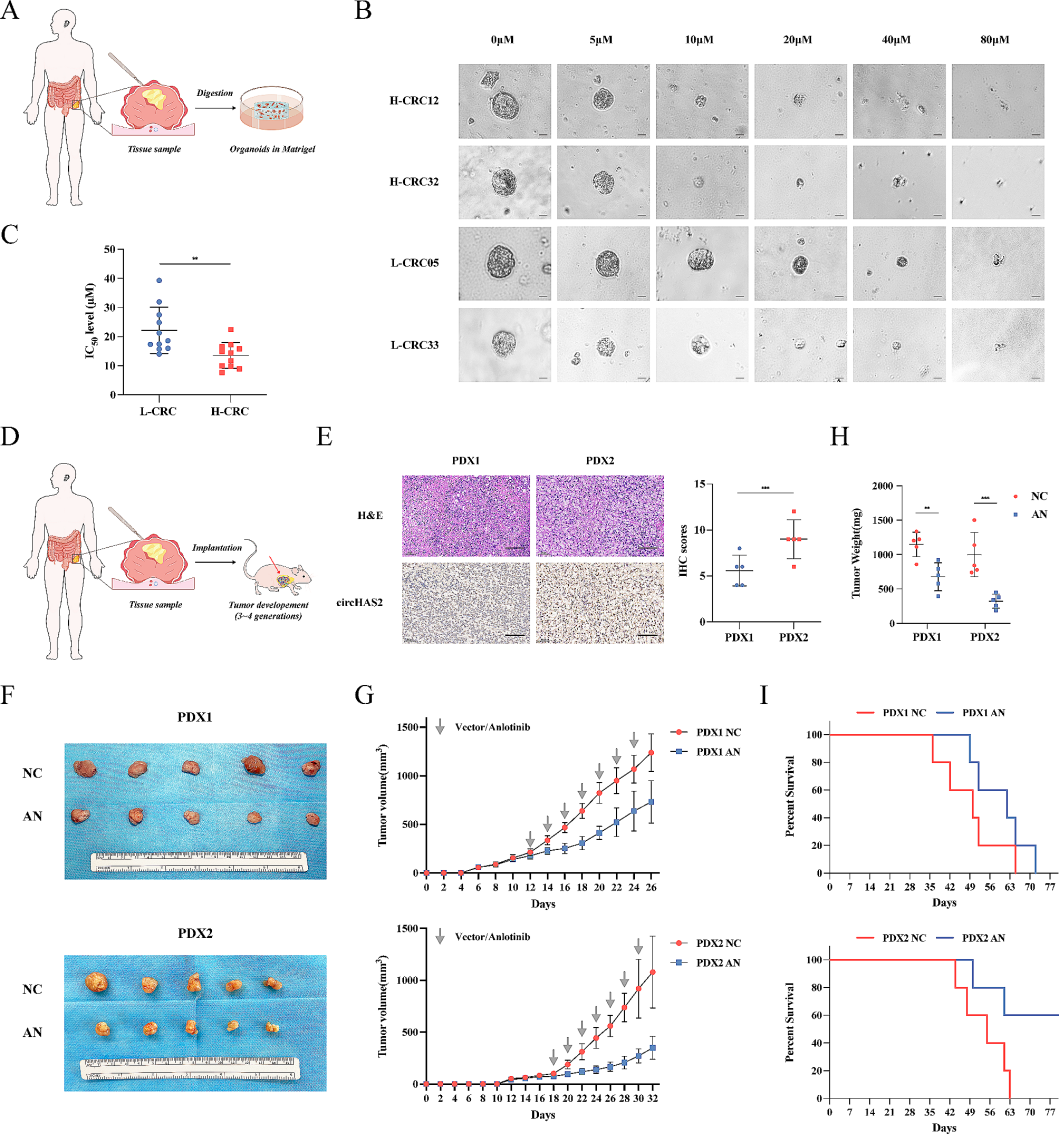
CircHAS2 as a predictive biomarker for response to anlotinib. **A** Schematic of the experimental setup for in vitro culture experiments involving PDO models. **B** Representative images of PDOs treated with the indicated concentrations of anlotinib via microscopy (scale bar: 10 μm). **C** The IC50 values of organoids treated with the indicated concentrations of anlotinib. **D** Schematic of the experimental setup for in vivo experiments involving PDX models. **E** Representative images and IHC scores of CRC PDX models were established and evaluated for circHAS2 expression by ISH staining. **F** Representative images of subcutaneous PDX tumors. The mice were given vehicle control and anlotinib, respectively. **G** The PDX tumors growth curve with measurements taken every two days. **H** The weights of subcutaneous PDX tumors were analyzed at the endpoint in each group. **I** Kaplan–Meier survival curves of the PDX models after the indicated treatments. Statistical significance in two-group experiments was evaluated using a two-sided Student’s t-test. Survival analysis was performed using the Log-rank method. Data are represented as mean ± SD; *, *P* < 0.05; **, *P* < 0.01; ***, *P* < 0.001; ns, no significance

To further confirm the effect of anlotinib on patients with CRC, we treated PDO cells with anlotinib, noting circHAS2’s influence on anlotinib sensitivity in 22 CRC patient-derived samples (Fig. 7A). When we divided the PDOs into two groups based on circHAS2 expression levels, the PDOs with high circHAS2 expression exhibited significant proliferation suppression under anlotinib, correlating with lower IC50 values. Monitoring PDO cell proliferation under various drug concentrations revealed that patients with high circHAS2 expression were more sensitive to anlotinib, while those with low circHAS2 expression exhibited higher IC50 values (Fig. 7B-C and S9A-B). These results suggest suggest circHAS2 as a potential marker for guiding anlotinib treatment in CRC. Subsequently, we conducted two patient-derived tumor xenograft (PDX) models and treated mice with anlotinib (Fig. 7D). Using immunohistochemical (IHC) score and gene expression analysis of patient tumor tissues, we determined that PDX2 expressed high levels of circHAS2, whereas PDX1 expressed lower levels (Fig. 7E and S9C). Anlotinib significantly reduced tumor growth in both PDX groups, particularly in high circHAS2-expressing tumors with minor effects in the PDX1 model (Fig. 7F-H). Furthermore, anlotinib treatment significantly prolonged the survival of mice with high circHAS2 expression (Fig. 7I). Thus, circHAS2 emerges as a key oncogenic factor in CRC and a potential biomarker for anlotinib sensitivity.

### Clinical response to anlotinib therapy based on circHAS2 expression

**Fig. 8:**
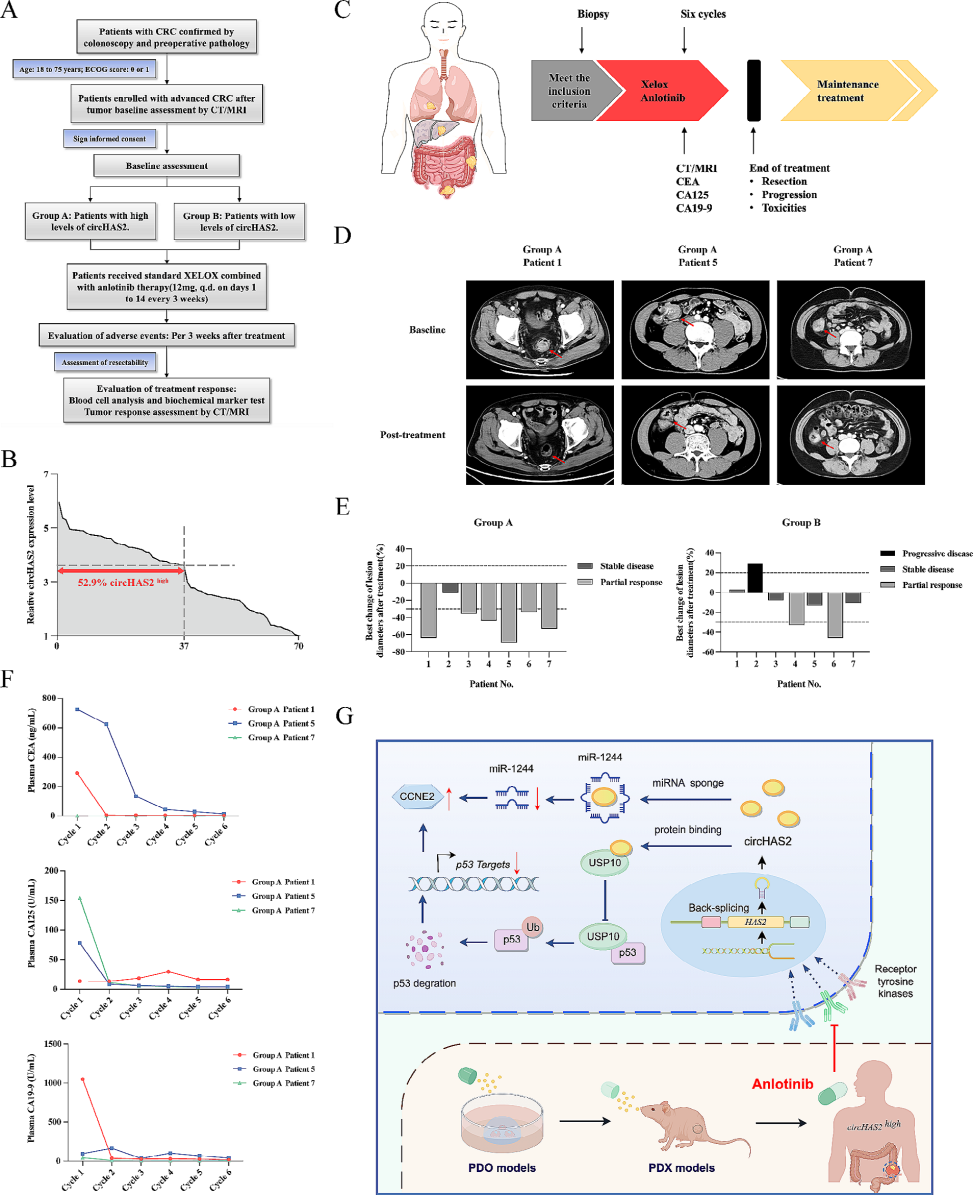
Responses of patients with CRC to anlotinib therapy. **A** The CONSORT diagram shows the patient flow through the clinical trial. **B** Distribution of circHAS2 expression levels in 70 patients with CRC as represented by relative expression level. **C** Diagram of the clinical trial design for the treatment of patients with advanced CRC. **D** Computed tomography scans of three representative CRC patients collected at baseline and after anlotinib treatment. The lesions were significantly reduced after treatment. **E** The best tumor response and maximum percent change from baseline were in the sum of target-lesion diameters during anlotinib treatment in an individual patient according to RECIST v1.1. **F** Serum CEA, CA125, and CA19-9 level of Group A (patient1,5, and 7) during the indicated treatment. **G** Schematic diagram of circHAS2 activating CCNE2 to promote cell proliferation and anlotinib sensitivity in colorectal cancer by Figdraw (www.figdraw.com)

Our findings suggest a specific benefit of anlotinib in CRC patients with high circHAS2 levels. A clinical study (ALTER-C001) demonstrated that anlotinib combined with the XELOX regimen for first-line and maintenance therapy had a modest benefit for patients with metastatic CRC [34]. Based on our preclinical data, we initiated a clinical trial at Ruijin Hospital (ClinicalTrials.gov identifier: NCT05262335) to evaluate the efficacy and safety of anlotinib plus the XELOX regimen in patients with advanced gastrointestinal tumors. CircHAS2 expression levels were employed as a stratification criterion to explore treatment responses in advanced CRC patients (Fig. 8A). Utilizing a tissue sample library comprising 70 CRC specimens, 52.9% of CRC cases exhibited high circHAS2 levels (Fig. 8B). Non-invasive imaging and serum tumor marker evaluations were conducted before, during, and after anlotinib-based chemotherapy to assess treatment responses (Fig. 8C). Currently, 14 patients with advanced CRC who have received treatment, 7 were categorized as having high circHAS2 expression (Fig. S9D). The clinical information of patients and genetic mutation details has been compiled (Fig. S9E and Table S4). Notably, computed tomography scans revealed a substantial reduction in the primary tumor size in the group A cohort (Fig. 8D). Compared to the low circHAS2-expressing group B cohort, group A displayed higher tumor response rates, with a Disease Control Rate (DCR) of 100.0% and an Overall Response Rate (ORR) of 57.1% (Fig. 8E). An overall incidence rate of 71.4% (10/14) for all-grade treatment-emergent adverse events (TEAEs) and a rate of 28.5% for ≥ 3 grade TEAEs. The major events included a decrease in neutrophil count (14.2%), a decrease in platelet count (14.2%), hand-foot skin reaction (14.2%), and hypertension (7.1%). Notably, reductions in serum tumor markers CEA, CA125, and CA19-9 were closely associated with favorable prognoses in CRC [35], especially in specific group A patients, aligning with favorable prognoses (Fig. 8F). In that sense, our preliminary PDO and PDX models displayed strong therapeutic responsiveness, serving as robust proxies for human conditions. Given that reductions in tumor burden closely correlate with patient survival prognoses, our initial clinical responses may portend favorable long-term survival outcomes.

## Discussion

Among de novo CRC patients, 20% have metastatic disease upon diagnosis, and 25% with localized disease would develop metastasis [36]. Targeted therapies, like multi-target TKIs, are important in treating advanced CRC [4, 37]. Among 42 kinds of TKIs with demonstrated preclinical anti-tumor efficacy, only certain patients are sensitive to the drugs [38]. To tackle the dilemma in the clinical translation of targeted therapies, researchers have been exploring the roles of ncRNAs in drug resistance mechanisms and biomarkers [39]. For instance, Li et al. delineated the dysregulation of ncRNAs in EGFR TKI-resistant lung cancer, discussing clinical applications as therapeutic biomarkers [40]. Additional research by Dong et al. suggested that lncGAS5 could serve as a potential biomarker, and its overexpression could reverse resistance to gefitinib [41]. On the other hand, scholars propose utilizing biomarkers to identify patients who are sensitive to TKIs. A study by Sun et al. reported that aberrant expression patterns of circFBXW7 dictated cellular responses to osimertinib [42]. Therefore, the rational stratification of patients to identify sensitive subpopulations is crucial.

In our study, we firstly found circHAS2, a circRNA of ncRNAs, is significantly down-regulated in an anlotinib-concentration-dependent manner. Simultaneously, circHAS2 is markedly up-regulated in the CRC tissues, while little expressed in adjacent non-cancerous tissues. It is noteworthy that patients with high circHAS2 expression have a worse prognosis, indicating that circHAS2 can serve as a novel diagnostic biomarker for CRC patients. In vitro and in vivo experiments reveal that circHAS2 promotes CRC proliferation significantly. However, the precise mechanism by which anlotinib modulates the degradation of circHAS2 remains unclear. We hypothesize that anlotinib may induce an increase in RNase H1 following tyrosine kinase activation, leading to the degradation of RNA strands within R-loops [15]. As a focus of our subsequent research, this intriguing possibility warrants further investigation.

Additionally, consistent with Ju et al. report, our study demonstrates that circHAS2 is predominantly localized in the cytoplasm [43]. It acts as a sponge for miR-1244, up-regulating the downstream expression of CCNE2 and thus promoting the cell cycle of CRC cells. However, rescue experiments reveal that miR-1244 cannot fully restore the regulatory effect of circHAS2 on CCNE2 expression. Meanwhile, the research also identifies a novel mechanism that circHAS2 physically interacts with USP10 to disrupt the binding of USP10 and p53. USP10, an important member of the ubiquitin-specific protease family, is widely expressed in the cytoplasm and nucleus of almost all cells, and has been linked to anti-cancer activities [33, 44, 45]. As a deubiquitinase, USP10 prevents the target protein p53 from interacting with ubiquitin, blocking the polyubiquitination and subsequent degradation of p53 [33]. Reduced p53 protein results in decreased expression of downstream target gene p21 and increased levels of CCNE2, promoting cell cycle acceleration and proliferation. However, little research has been conducted on the complete mechanism of circHAS2 in CRC. This bidirectional pathway elucidates the specific cellular processes modulated by anlotinib-regulated circHAS2 in CRC.

Currently, to enhance the predictability of drug clinical efficacy, complex preclinical models are being employed [46, 47]. . Increasing evidence suggests that PDO and PDX models can be used in parallel or sequential with clinical trials to evaluate the effectiveness of drugs on patients, replacing traditional preclinical studies in animals [48]. A PDO library of 22 CRC patients and PDX models were used in the present study, which revealed that CRC patients with high levels of circHAS2 expression were more likely to benefit from targeted therapy with anlotinib. However, only a few biomarkers have been validated and integrated into clinical practice [49]. In this context, combining PDO and PDX models from translational research may increase the possibility of developing novel molecular biomarkers [50–52]. This paves the way for translating new biomarkers and targets from the laboratory to personalized treatment.

Clinical trials over the past five years have demonstrated that individualized treatment based on molecular and pathological characteristics of tumors can improve overall survival rates [53]. However, larger-scale and multicenter trials are still needed to confirm their benefits. In our study, based on the evidence provided by preclinical PDO and PDX models, anlotinib-based prospective Phase II clinical trial was commenced (ClinicalTrials.gov identifier: NCT05262335). Patients expressing high levels of circHAS2 yielded meaningful clinical remission, with a DCR of 100.0% and an ORR of 85.7%. Our findings offered a promising targeted strategy for approximately 52.9% of advanced CRC patients who had high circHAS2 levels. Furthermore, anlotinib was also well-tolerated, with no observed serious adverse events.

## Conclusion

In summary, the present study reveals the inhibitory effect of anlotinib in CRC. CircHAS2 not only up-regulates CCNE2 through the traditional ceRNA mechanism, but also disrupts the binding between USP10 and p53, thereby increasing CCNE2 expression. This could provide a comprehensive profile for the bidirectional modulatory role of anlotinib-regulated circHAS2 in suppressing CRC proliferation. Patients with high circHAS2 expression are more sensitive to anlotinib, making it a potential molecular biomarker for targeted therapy.

### Electronic supplementary material

Below is the link to the electronic supplementary material.

Supplementary Material 1

Supplementary Material 2

Supplementary Material 3

Supplementary Material 4

Supplementary Material 5

Supplementary Material 6

Supplementary Material 7

## Data Availability

The data that support the findings of this study are available from the corresponding author upon reasonable request.

## Abbreviations

CRC: Colorectal cancer
circRNAs: Circular RNAs
ncRNAs: Noncoding RNAs
TKIs: Tyrosine kinase inhibitors
RTK: Receptor tyrosine kinases
USP10: Ubiquitin specific peptidase 10
H&E: Hematoxylin and eosin
FISH: RNA fluorescence in situ hybridization
ISH: In situ hybridization
IP: Immunoprecipitation
RIP: RNA immunoprecipitation
TNM: Tumor-node-metastasis
ceRNAs: Competitive endogenous RNAs
RBPs: RNA-binding proteins
DCR: Disease Control Rate
ORR: Overall Response Rate
PDO: Patient-derived organoids
PDX: Patient-derived tumor xenograft

## References

[1] Siegel RL, Miller KD, Fuchs HE, Jemal A (2022). Cancer statistics, 2022. CA Cancer J Clin.

[2] Sung H, Ferlay J, Siegel RL, Laversanne M, Soerjomataram I, Jemal A, Bray F (2021). Global Cancer statistics 2020: GLOBOCAN estimates of incidence and Mortality Worldwide for 36 cancers in 185 countries. CA Cancer J Clin.

[3] Jin Z, Sinicrope FA (2022). Mismatch repair-deficient Colorectal Cancer: building on checkpoint blockade. J Clin Oncol.

[4] Messersmith WA (2019). NCCN guidelines updates: management of metastatic colorectal Cancer. J Natl Compr Canc Netw.

[5] Ferguson FM, Gray NS (2018). Kinase inhibitors: the road ahead. Nat Rev Drug Discov.

[6] Li H, Huang H, Zhang T, Feng H, Wang S, Zhang Y, Ji X, Cheng X, Zhao R (2022). Apatinib: a Novel Antiangiogenic Drug in Monotherapy or Combination Immunotherapy for Digestive System malignancies. Front Immunol.

[7] Xie C, Wan X, Quan H, Zheng M, Fu L, Li Y, Lou L (2018). Preclinical characterization of anlotinib, a highly potent and selective vascular endothelial growth factor receptor-2 inhibitor. Cancer Sci.

[8] Shen G, Zheng F, Ren D, Du F, Dong Q, Wang Z, Zhao F, Ahmad R, Zhao J (2018). Anlotinib: a novel multi-targeting tyrosine kinase inhibitor in clinical development. J Hematol Oncol.

[9] Huang NS, Wei WJ, Xiang J, Chen JY, Guan Q, Lu ZW, Ma B, Sun GH, Wang YL, Ji QH, Wang Y (2021). The efficacy and safety of Anlotinib in Neoadjuvant Treatment of locally advanced thyroid Cancer: a single-arm phase II clinical trial. Thyroid.

[10] Long Z, Lu Y, Li M, Fu Z, Akbar Y, Li J, Chen G, Zhang HM, Wang Q, Xiang L, Wang Z. Evaluation of Anlotinib Combined with Adriamycin and Ifosfamide as Conversion Therapy for Unresectable Soft Tissue Sarcomas. Cancers (Basel) 2023, 15. 10.3390/cancers15030700 PMC9913396 36765658

[11] Engreitz JM, Haines JE, Perez EM, Munson G, Chen J, Kane M, McDonel PE, Guttman M, Lander ES (2016). Local regulation of gene expression by lncRNA promoters, transcription and splicing. Nature.

[12] Lu S, Zhang J, Lian X, Sun L, Meng K, Chen Y, Sun Z, Yin X, Li Y, Zhao J (2019). A hidden human proteome encoded by ‘non-coding’ genes. Nucleic Acids Res.

[13] Saw PE, Xu X, Chen J, Song EW (2021). Non-coding RNAs: the new central dogma of cancer biology. Sci China Life Sci.

[14] Liu CX, Chen LL (2022). Circular RNAs: characterization, cellular roles, and applications. Cell.

[15] Liu CX, Li X, Nan F, Jiang S, Gao X, Guo SK, Xue W, Cui Y, Dong K, Ding H (2019). Structure and degradation of circular RNAs regulate PKR activation in Innate Immunity. Cell.

[16] Dong W, Dai ZH, Liu FC, Guo XG, Ge CM, Ding J, Liu H, Yang F (2019). The RNA-binding protein RBM3 promotes cell proliferation in hepatocellular carcinoma by regulating circular RNA SCD-circRNA 2 production. EBioMedicine.

[17] Wang L, Yi J, Lu LY, Zhang YY, Wang L, Hu GS, Liu YC, Ding JC, Shen HF, Zhao FQ (2021). Estrogen-induced circRNA, circPGR, functions as a ceRNA to promote estrogen receptor-positive breast cancer cell growth by regulating cell cycle-related genes. Theranostics.

[18] He D, Yang X, Kuang W, Huang G, Liu X, Zhang Y (2020). The Novel Circular RNA Circ-PGAP3 promotes the Proliferation and Invasion of Triple negative breast Cancer by regulating the miR-330-3p/Myc Axis. Onco Targets Ther.

[19] Zhong W, Yu Z, Zhan J, Yu T, Lin Y, Xia ZS, Yuan YH, Chen QK (2015). Association of serum levels of CEA, CA199, CA125, CYFRA21-1 and CA72-4 and disease characteristics in colorectal cancer. Pathol Oncol Res.

[20] Sanders DS, Kerr MA (1999). Lewis blood group and CEA related antigens; coexpressed cell-cell adhesion molecules with roles in the biological progression and dissemination of tumours. Mol Pathol.

[21] Hewish M, Lord CJ, Martin SA, Cunningham D, Ashworth A (2010). Mismatch repair deficient colorectal cancer in the era of personalized treatment. Nat Rev Clin Oncol.

[22] Sinicrope FA (2010). DNA mismatch repair and adjuvant chemotherapy in sporadic colon cancer. Nat Rev Clin Oncol.

[23] Vilar E, Gruber SB (2010). Microsatellite instability in colorectal cancer-the stable evidence. Nat Rev Clin Oncol.

[24] Jagtap U, Anderson ES, Slack FJ (2023). The emerging value of circular noncoding RNA research in Cancer diagnosis and treatment. Cancer Res.

[25] Eisenhauer EA, Therasse P, Bogaerts J, Schwartz LH, Sargent D, Ford R, Dancey J, Arbuck S, Gwyther S, Mooney M (2009). New response evaluation criteria in solid tumours: revised RECIST guideline (version 1.1). Eur J Cancer.

[26] Bertrand E, Chartrand P, Schaefer M, Shenoy SM, Singer RH, Long RM (1998). Localization of ASH1 mRNA particles in living yeast. Mol Cell.

[27] Zheng R, Zhang K, Tan S, Gao F, Zhang Y, Xu W, Wang H, Gu D, Zhu L, Li S (2022). Exosomal circLPAR1 functions in colorectal cancer diagnosis and tumorigenesis through suppressing BRD4 via METTL3-eIF3h interaction. Mol Cancer.

[28] Cheng X, Jin Z, Ji X, Shen X, Feng H, Morgenlander W, Ou B, Wu H, Gao H, Ye F (2019). ETS variant 5 promotes colorectal cancer angiogenesis by targeting platelet-derived growth factor BB. Int J Cancer.

[29] Salmena L, Poliseno L, Tay Y, Kats L, Pandolfi PP (2011). A ceRNA hypothesis: the Rosetta Stone of a hidden RNA language?. Cell.

[30] Riley D, Carragher NO, Frame MC, Wyke JA (2001). The mechanism of cell cycle regulation by v-Src. Oncogene.

[31] Li X, Yang L, Chen LL (2018). The Biogenesis, functions, and challenges of Circular RNAs. Mol Cell.

[32] Yang R, Chen H, Xing L, Wang B, Hu M, Ou X, Chen H, Deng Y, Liu D, Jiang R, Chen J (2022). Hypoxia-induced circWSB1 promotes breast cancer progression through destabilizing p53 by interacting with USP10. Mol Cancer.

[33] Yuan J, Luo K, Zhang L, Cheville JC, Lou Z (2010). USP10 regulates p53 localization and stability by deubiquitinating p53. Cell.

[34] He J, Liu Y, Liu C, Hu H, Sun L, Xu D, Li J, Wang J, Chen X, Lin R (2023). A Randomized Phase III Study of Anlotinib Versus Bevacizumab in Combination with CAPEOX as First-Line Therapy for RAS/BRAF Wild-Type metastatic colorectal Cancer: a clinical trial protocol. Technol Cancer Res Treat.

[35] Li C, Zhao K, Zhang D, Pang X, Pu H, Lei M, Fan B, Lv J, You D, Li Z, Zhang T (2023). Prediction models of colorectal cancer prognosis incorporating perioperative longitudinal serum tumor markers: a retrospective longitudinal cohort study. BMC Med.

[36] Biller LH, Schrag D (2021). Diagnosis and treatment of metastatic colorectal Cancer: a review. JAMA.

[37] Shin AE, Giancotti FG, Rustgi AK (2023). Metastatic colorectal cancer: mechanisms and emerging therapeutics. Trends Pharmacol Sci.

[38] Grothey A, Van Cutsem E, Sobrero A, Siena S, Falcone A, Ychou M, Humblet Y, Bouche O, Mineur L, Barone C (2013). Regorafenib monotherapy for previously treated metastatic colorectal cancer (CORRECT): an international, multicentre, randomised, placebo-controlled, phase 3 trial. Lancet.

[39] Cohen P, Cross D, Janne PA (2021). Kinase drug discovery 20 years after imatinib: progress and future directions. Nat Rev Drug Discov.

[40] Li J, Li P, Shao J, Liang S, Wan Y, Zhang Q, Li C, Li Y, Wang C. Emerging role of noncoding RNAs in EGFR TKI-Resistant Lung Cancer. Cancers (Basel) 2022, 14. 10.3390/cancers14184423 PMC9496789 36139582

[41] Dong S, Qu X, Li W, Zhong X, Li P, Yang S, Chen X, Shao M, Zhang L (2015). The long non-coding RNA, GAS5, enhances gefitinib-induced cell death in innate EGFR tyrosine kinase inhibitor-resistant lung adenocarcinoma cells with wide-type EGFR via downregulation of the IGF-1R expression. J Hematol Oncol.

[42] Li K, Peng ZY, Wang R, Li X, Du N, Liu DP, Zhang J, Zhang YF, Ma L, Sun Y (2023). Enhancement of TKI sensitivity in lung adenocarcinoma through m6A-dependent translational repression of wnt signaling by circ-FBXW7. Mol Cancer.

[43] Ma S, Gu X, Shen L, Chen Y, Qian C, Shen X, Ju S (2021). CircHAS2 promotes the proliferation, migration, and invasion of gastric cancer cells by regulating PPM1E mediated by hsa-miR-944. Cell Death Dis.

[44] Lim R, Sugino T, Nolte H, Andrade J, Zimmermann B, Shi C, Doddaballapur A, Ong YT, Wilhelm K, Fasse JWD (2019). Deubiquitinase USP10 regulates notch signaling in the endothelium. Science.

[45] Lu C, Ning Z, Wang A, Chen D, Liu X, Xia T, Tekcham DS, Wang W, Li T, Liu X (2018). USP10 suppresses tumor progression by inhibiting mTOR activation in hepatocellular carcinoma. Cancer Lett.

[46] Vlachogiannis G, Hedayat S, Vatsiou A, Jamin Y, Fernandez-Mateos J, Khan K, Lampis A, Eason K, Huntingford I, Burke R (2018). Patient-derived organoids model treatment response of metastatic gastrointestinal cancers. Science.

[47] Ganesh K, Wu C, O’Rourke KP, Szeglin BC, Zheng Y, Sauve CG, Adileh M, Wasserman I, Marco MR, Kim AS (2019). A rectal cancer organoid platform to study individual responses to chemoradiation. Nat Med.

[48] Wadman M (2023). FDA no longer has to require animal testing for new drugs. Science.

[49] Yao Y, Xu X, Yang L, Zhu J, Wan J, Shen L, Xia F, Fu G, Deng Y, Pan M (2020). Patient-derived Organoids Predict Chemoradiation responses of locally advanced rectal Cancer. Cell Stem Cell.

[50] Letai A, Bhola P, Welm AL (2022). Functional precision oncology: testing tumors with drugs to identify vulnerabilities and novel combinations. Cancer Cell.

[51] Macias RIR, Cardinale V, Kendall TJ, Avila MA, Guido M, Coulouarn C, Braconi C, Frampton AE, Bridgewater J, Overi D (2022). Clinical relevance of biomarkers in cholangiocarcinoma: critical revision and future directions. Gut.

[52] Pamarthy S, Sabaawy HE (2021). Patient derived organoids in prostate cancer: improving therapeutic efficacy in precision medicine. Mol Cancer.

[53] Wensink E, Bond M, Kucukkose E, May A, Vink G, Koopman M, Kranenburg O, Roodhart J (2021). A review of the sensitivity of metastatic colorectal cancer patients with deficient mismatch repair to standard-of-care chemotherapy and monoclonal antibodies, with recommendations for future research. Cancer Treat Rev.

