# Supplementary material for: CircHAS2 activates CCNE2 to promote cell proliferation and sensitizes the response of colorectal cancer to anlotinib
Source: Mol Cancer. 2024 Mar 21;23:59. doi: 10.1186/s12943-024-01971-7 (PMC10956180; doi:10.1186/s12943-024-01971-7)
Supplement: Supplementary file 1 — Supplementary Material 1 [file 12943_2024_1971_MOESM1_ESM.pdf]

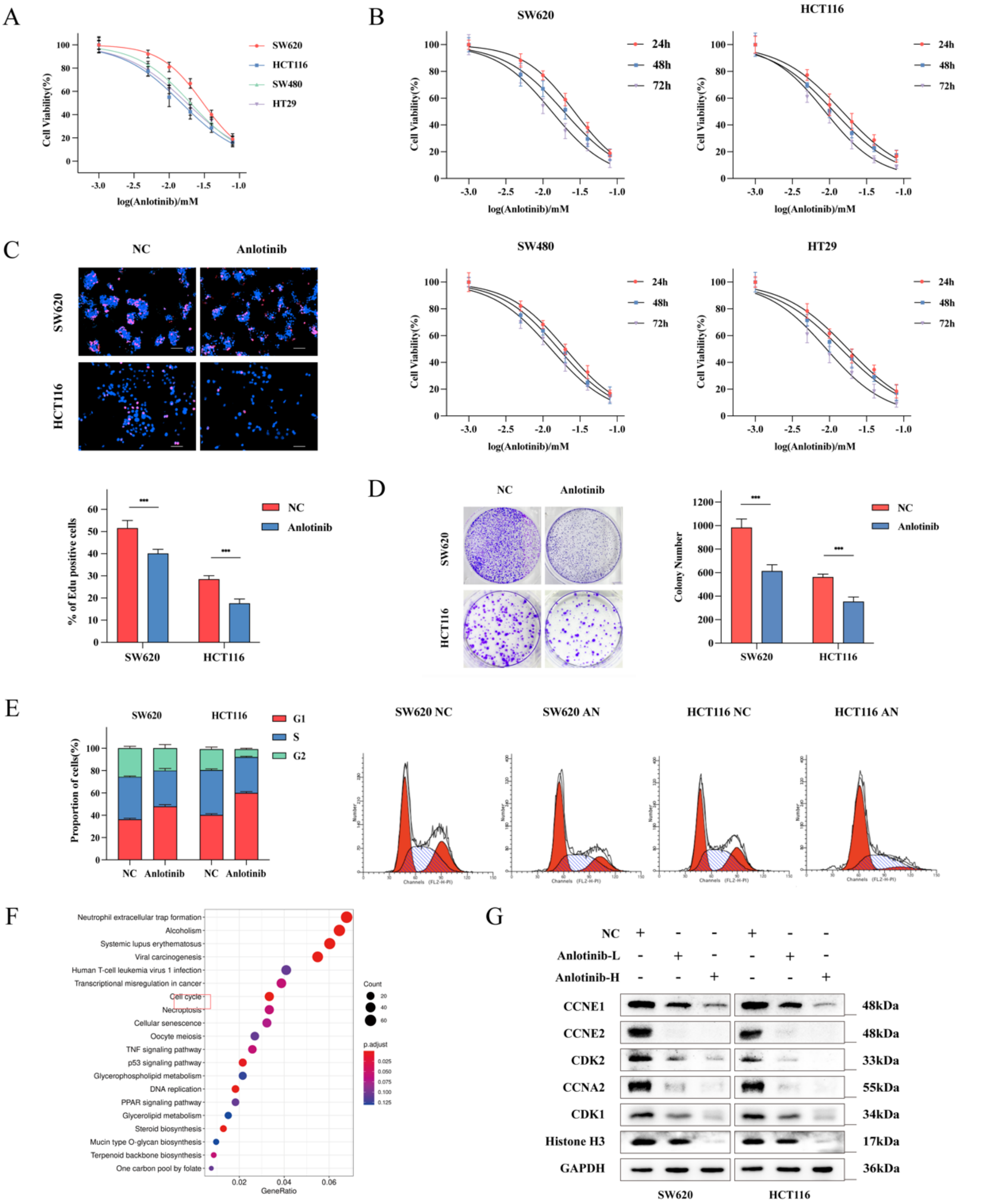

Figure S1: Anlotinib showing therapeutic effects in vitro. A The CCK-8 assay was used to detect the effect of different concentrations of anlotinib on CRC cell viability in SW620, HCT116, SW480 and HT29 cells. **B** The CCK-8 assay was employed to assess the impact of varying concentrations and exposure durations of anlotinib on the viability of SW620, HCT116, SW480 and HT29 cells. **C** Quantitative results and representative images of cell proliferation as measured by EdU incorporation after a

24-hour treatment (scale bar: 50 μm) treatment with anlotinib (10 μM) in SW620, HCT116 cells. **D** Quantitative results and representative cell proliferation images were determined by colony formation assay after two weeks of treatment with anlotinib (2 μM) in SW620, HCT116 cells. **E** The percentage of cells in the G1, S, and G2 phases of the entire cell population was determined by flow cytometry after a 24-hour treatment with anlotinib (10 μM) in SW620, HCT116 cells. Representative images of the flow cytometry analysis, illustrating the percentage of cells. **F** The results of the Kyoto Encyclopedia of Genes and Genomes (KEGG) pathway analysis with mRNA sequencing data in SW620 after a 24-hour treatment with vehicle control and anlotinib (10 μM). **G** Representative Western blotting images for the indicated proteins after a 24-hour treatment with anlotinib (L: 5 μM, H: 10 μM) treatment. GAPDH was used as an internal control. Statistical differences in qPCR, gene expression, clone formation, and EdU experiments were assessed using two-sided Student's t-test. Cell viability was determined by CCK-8 assay, employing two-way analysis of variance. Data are represented as mean ± SD; *, $P < 0.05$; **, $P < 0.01$; ***, $P < 0.001$; ns, no significance.

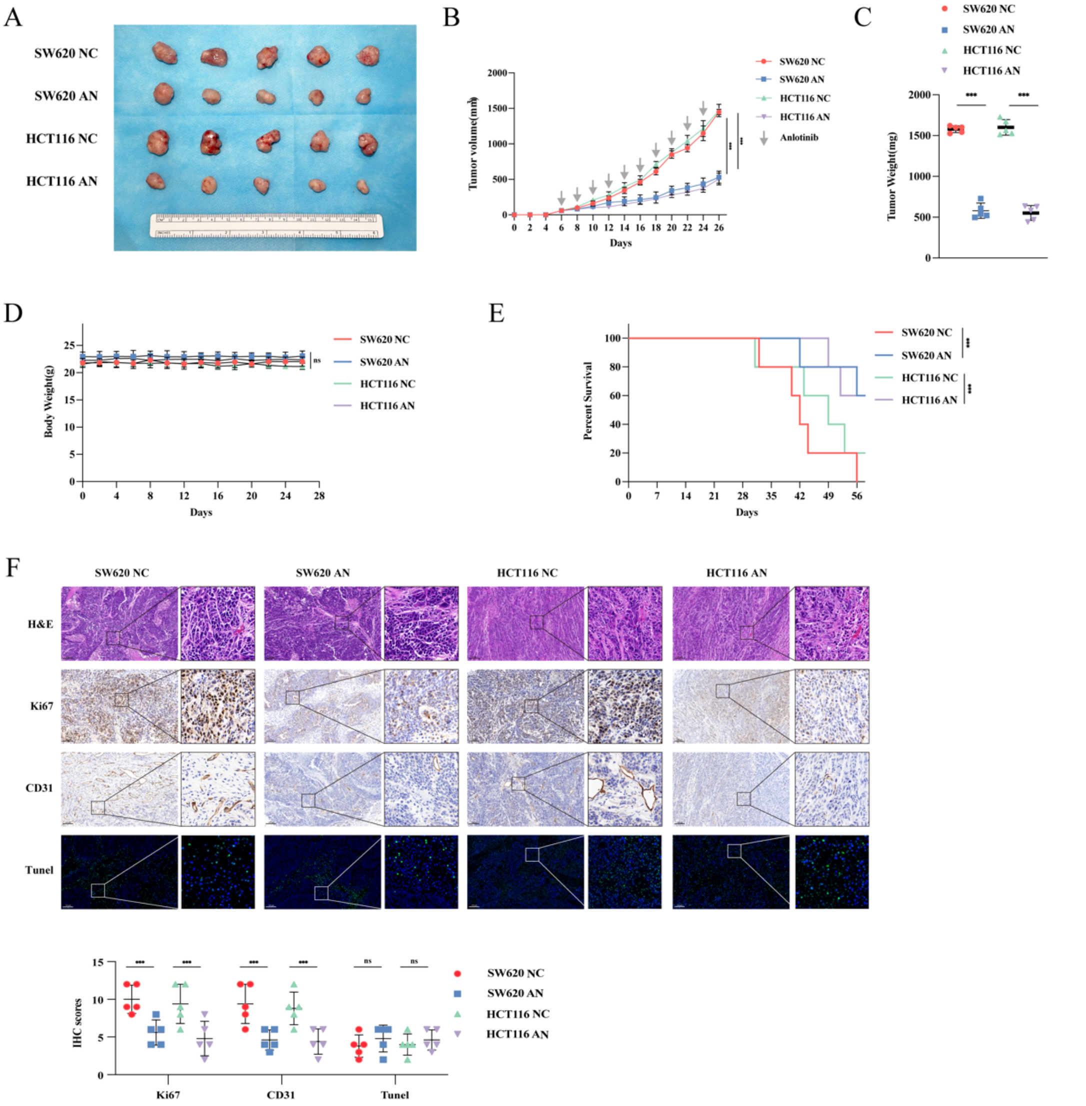

Figure S2: The efficacy of anlotinib in vivo. A Images of murine subcutaneous tumors (n=5). The mice were administered either a vehicle control or anlotinib (3 mg/kg, orally, every two days). **B** Tumor volumes were measured every two days. **C** Tumor weights were examined at the endpoint. **D** The body weights of mice were assessed every two days. **E** Kaplan–Meier survival curves of the mice after treatment. **F** Representative images and IHC scores of HE staining, IHC staining of Ki-67, CD31, and TUNEL assay of tumors in indicated treatment groups (scale bar: 100 μm). Details were magnified at a 5× scale. Statistical significance in two-group experiments was evaluated using a two-

sided Student's t-test. One-way analysis of variance was employed for multiple-group comparisons. Survival analysis was performed using the Log-rank method. Data are represented as mean ± SD; *, $P < 0.05$; **, $P < 0.01$; ***, $P < 0.001$; ns, no significance.

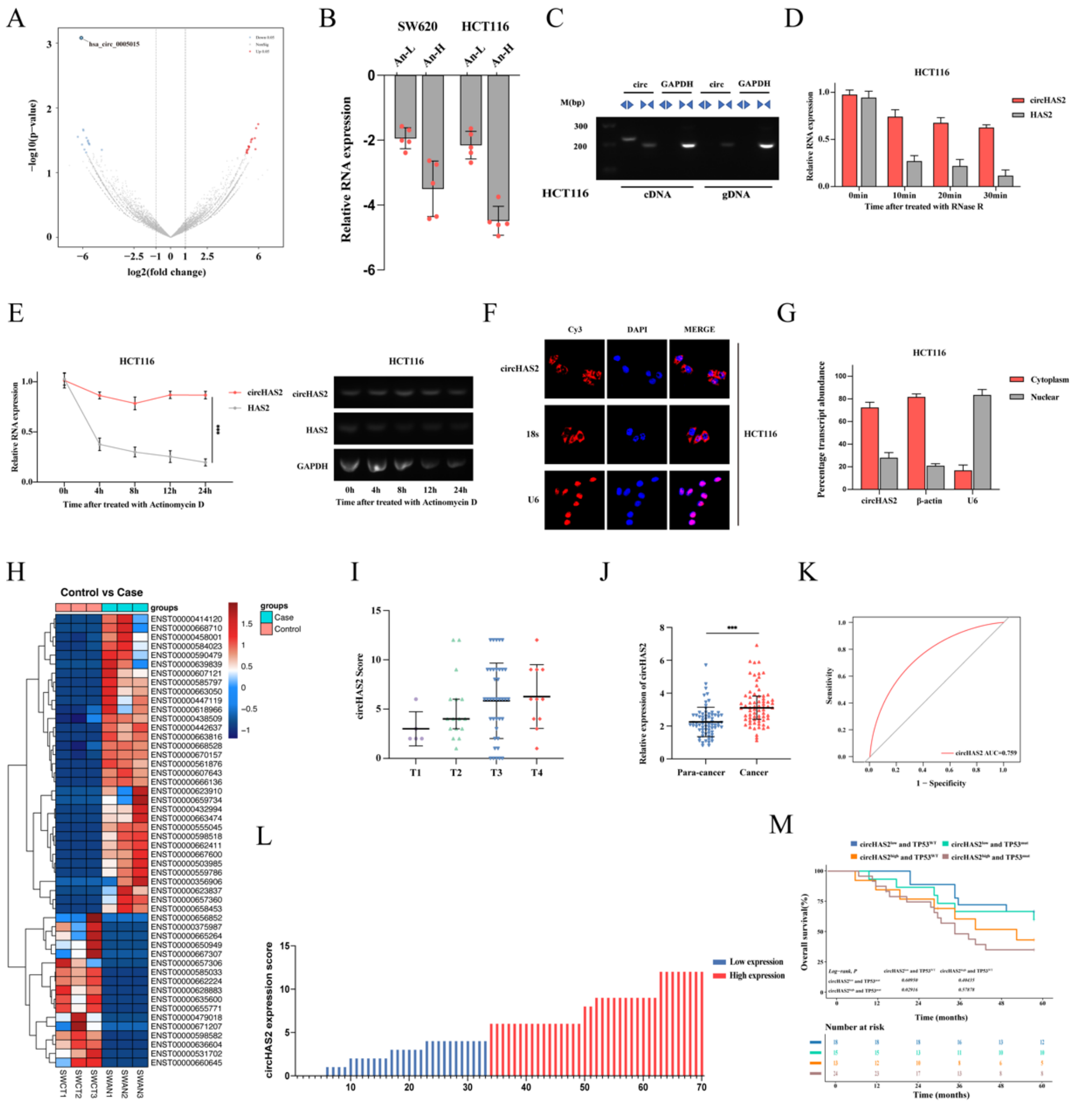

Figure S3 Anlotinib regulated-circHAS2 identified as a significant new target. A Volcano plot displaying notably differentially expressed circRNAs in SW620 cells after a 24-hour treatment with vehicle control and anlotinib (10 μM). **B** Relative expression of circHAS2 after anlotinib treatment (L: 5 μM, H: 10 μM) in SW620 and HCT116 cells for 24 hours. **C** PCR and agarose gel electrophoresis analysis in HCT116 cells verified that the divergent primers for circHAS2 could be amplified from cDNA but not gDNA. **D** Relative expression of circHAS2 and HAS2 mRNA after treatment with RNase R in HCT116 cells. **E** RNA abundance of circHAS2 and HAS2 mRNA after treatment with Actinomycin D in PCR and agarose gel electrophoresis analysis in

HCT116 cells. **F** Representative images of the FISH assay in HCT116 cells revealed circHAS2 predominantly located in the cytoplasm with the target probe labeled with Cy3 and nuclei stained with DAPI (scale bar: 20 μm). **H** Nuclear and cytoplasm fractionation assays in HCT116 cells showed the subcellular location of circHAS2. **H** Heatmap was generated to display the differential expression of lncRNAs in SW620 cells treated with either vehicle control and anlotinib (10 μM). **I** The expression of circHAS2 across different T stages was utilized by FISH. **J** The expression levels of circHAS2 in CRC tissues and paired normal tissues of microarrays were confirmed by qPCR. **K** The receiver operating characteristic (ROC) curves and the area under the curve (AUC) values were determined for circHAS2 alone. **L** The relative expression of circHAS2 was assessed in 70 CRC patients. A score of 4 or less was deemed indicative of low expression, while a score exceeding 4 was classified as high expression. **M** Overall survival curves of 70 CRC patients based on circHAS2 expression levels and p53 status in our center. Statistical significance in two-group experiments was evaluated using a two-sided Student's t-test. One-way analysis of variance was employed for multiple-group comparisons. Survival analysis was performed using the Log-rank method. Data are represented as mean ± SD; *, $P < 0.05$; **, $P < 0.01$; ***, $P < 0.001$; ns, no significance.

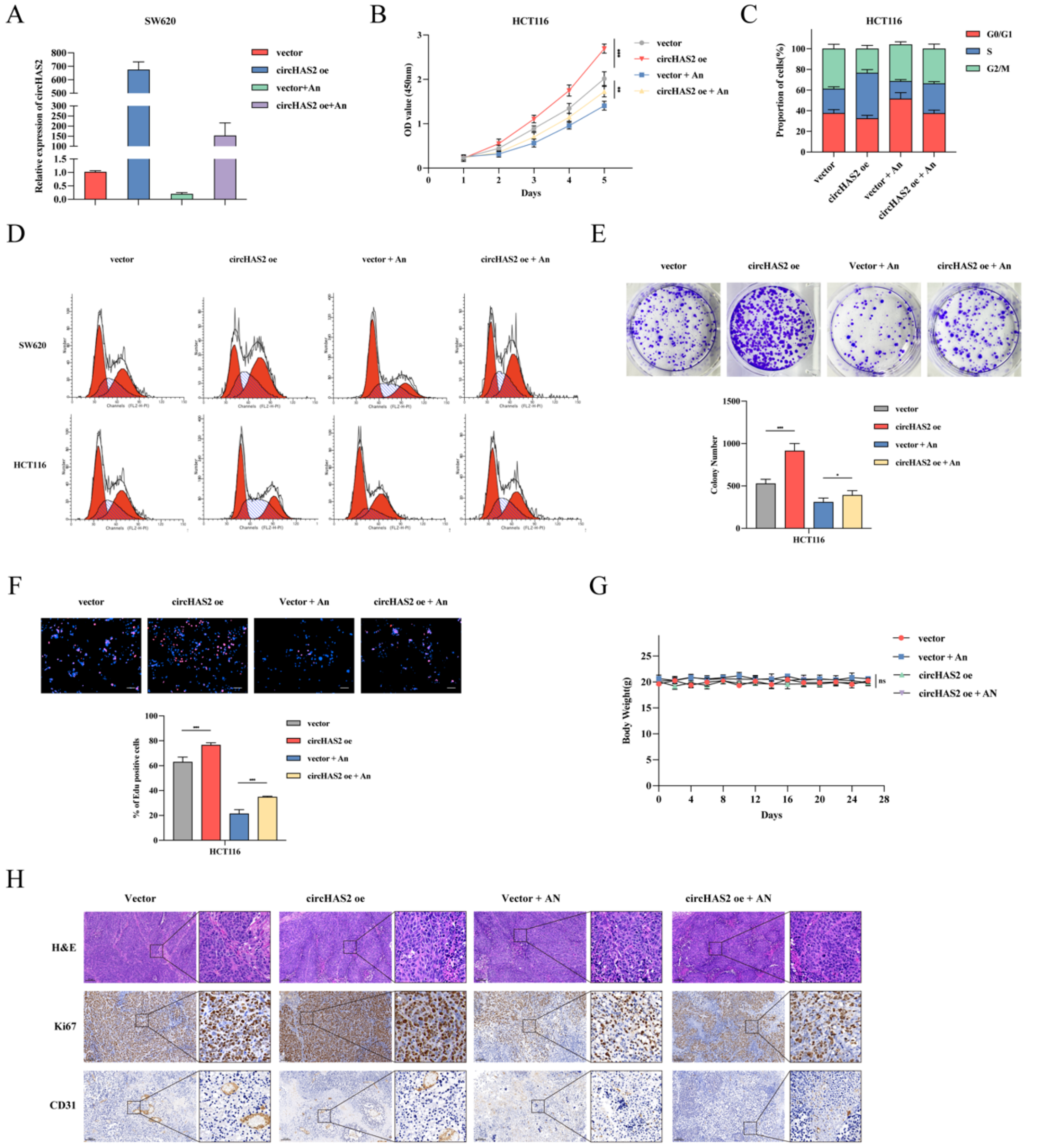

Figure S4 Oncogene circHAS2 can be rescued by anlotinib. A Relative expressions of circHAS2 in SW620 cells were verified with indicated treatment. **B** The viabilities of SW620/vector, SW620/circHAS2 oe with the addition of vehicle control or anlotinib cells were detected by CCK-8 assays in HCT116 cells. **C** The percentage of cells in the G1, S, and G2 phases of the whole cell population was determined by flow cytometry with indicated treatment in SW620 cells. **D** Representative images of the flow cytometry analysis, illustrating the percentage of cells in the G1, S, and G2/M phases of the cell cycle relative to the total cell population. **E** Quantitative results and

representative images of cell proliferation were conducted by colony formation assay with indicated treatment in HCT116 cells. **F** Quantitative results and representative images of cell proliferation were evaluated by EdU incorporation with indicated treatment in SW620 cells (scale bar: 50 μm) . **G** The body weights of mice were assessed every two days. **H** Representative images of HE staining and IHC staining (Ki-67 and CD31) assay of tumors of indicated treatment (scale bar: 100 μm). Details were magnified at a 5× scale. Statistical significance in two-group experiments was assessed using a two-sided Student's t-test, and multiple-group comparisons were conducted through one-way analysis of variance. Data are represented as mean ± SD; *, $P < 0.05$; **, $P < 0.01$; ***, $P < 0.001$; ns, no significance.

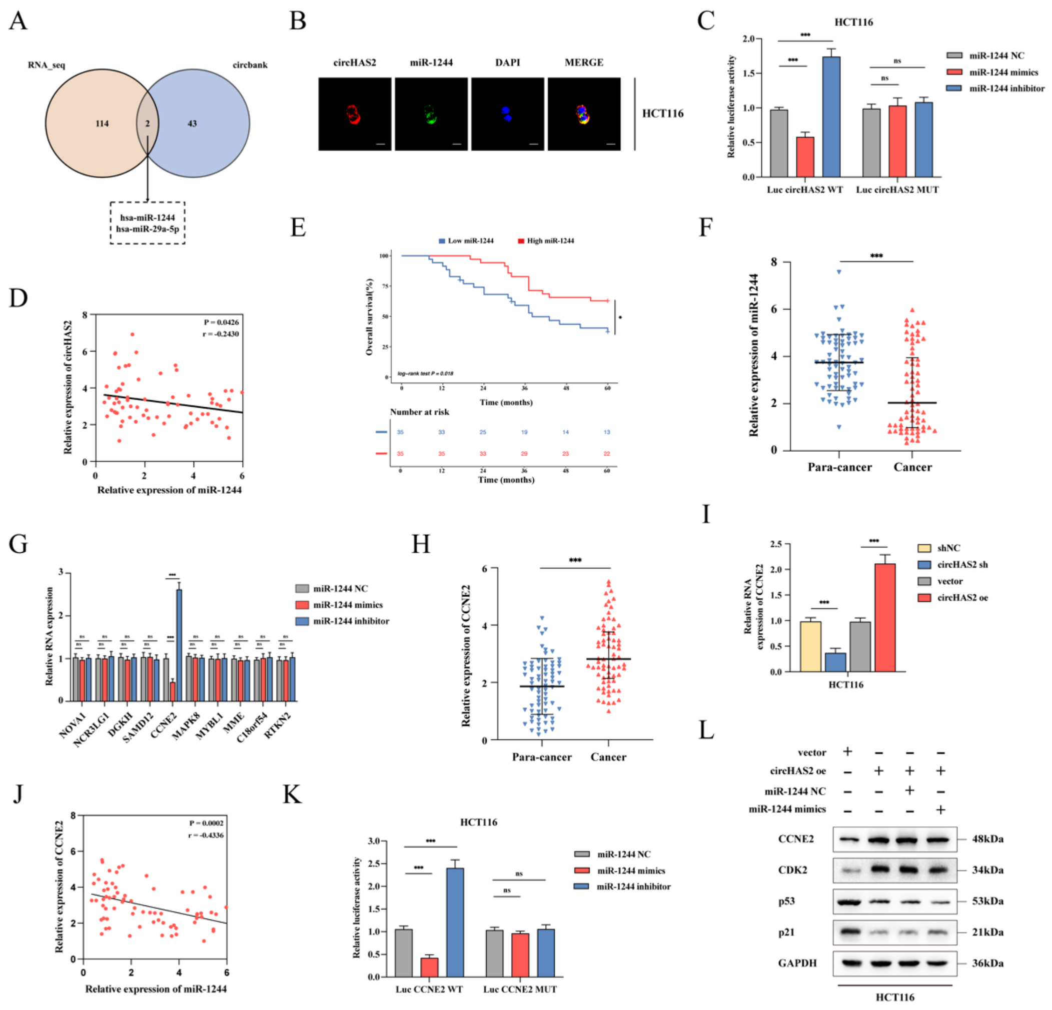

Figure S5 CircHAS2 serves as a miR-1244 sponge to regulate CCNE2 expression in CRC. A Prediction of potential downstream target genes for circHAS2 using RNA sequencing and circbank. **B** Representative images of the FISH assay revealed the colocalization of circHAS2 and miR-1244 in the cytoplasm of HCT116 cells with the target probe labeled with Cy3 and nuclei stained with DAPI (scale bar: 20 μm). **C** Luciferase activity of circHAS2 in HCT116 cells was verified with co-transfected Luc circHAS2 WT or MUT and miR-1244 mimics or inhibitor. **D** The correlation between circHAS2 and miR-1244 was determined by Pearson correlation analysis from our research center. **E** Overall survival curves of 70 CRC patients based on miR-1244 expression levels in our center. **F** The expression levels of miR-1244 in CRC tissues

and paired normal tissues of microarrays were confirmed by qPCR in our center. **G** Relative expressions of potential downstream target genes were confirmed in SW620 cells after indicated treatment. **H** The expression levels of CCNE2 in CRC tissues and paired normal tissues from microarrays were validated by qPCR in our center. **I** Relative CCNE2 expressions were verified after indicated treatment in HCT116 cells. **J** The correlation between CCNE2 and miR-1244 was determined by Pearson correlation analysis from our research center. **K** Luciferase activity of CCNE2 in HCT116 cells was verified with co-transfected Luc CCNE2 WT or MUT and miR-1244 mimics or inhibitor. **L** Representative Western blotting images in HCT116 cells with indicated treatments for CCNE2, CDK2, p53, and p21 proteins. GAPDH was used as an internal control. Statistical significance in two-group experiments was determined using a two-sided Student's t-test, and survival analysis was conducted using the Log-rank method. Data are represented as mean ± SD; *, $P < 0.05$; **, $P < 0.01$; ***, $P < 0.001$; ns, no significance.

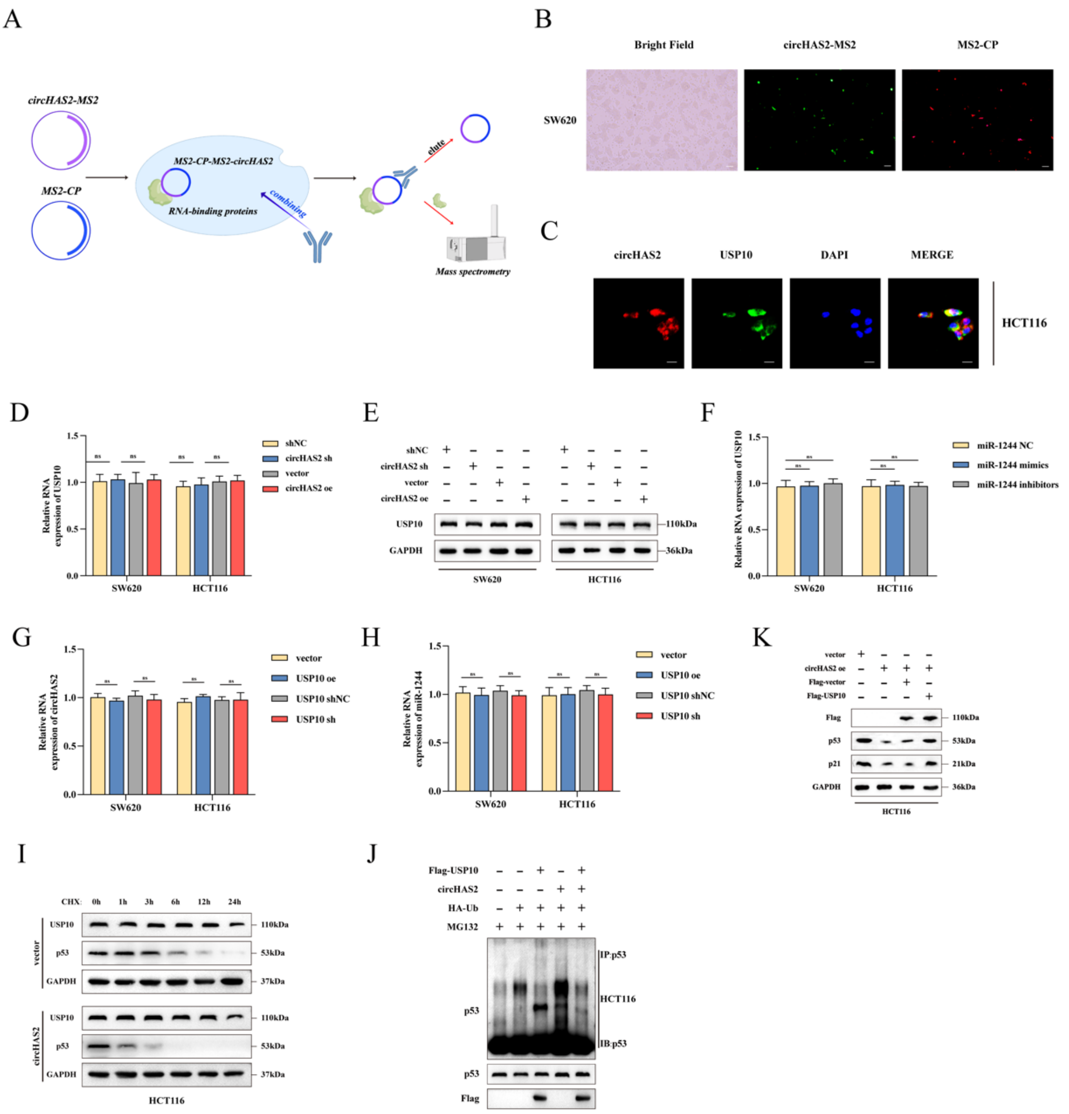

Figure S6 The interaction between circHAS2 and USP10 facilitates the process of p53 ubiquitination. A Schematic diagram of circHAS2 pull-down results using the MS2-tagging system. **B** Representative fluorescence images of co-transfection circHAS2-MS2 and MS2-CP into SW620 cells (scale bar: 50 μm). **C** Representative images of the FISH assay in HCT116 cells revealed the colocalization of circHAS2 and USP10 in the cytoplasm with the target probe labeled with Cy3 and nuclei stained with DAPI (scale bar: 20 μm). **D** Relative USP10 expressions were verified after indicated treatments in SW620 and HCT116 cells. **E** Representative Western blotting images with indicated treatments for USP10 proteins in SW620 and HCT116 cells. GAPDH was

used as an internal control. **F** Relative expressions of USP10 were confirmed after indicated treatment in SW620 and HCT116 cells. **G** Relative circHAS2 expressions were verified after indicated treatment in SW620 and HCT116 cells. **H** Relative expressions of miR-1244 were confirmed after indicated treatment in SW620 and HCT116 cells. **I** After indicated treatments were treated with various durations of cycloheximide (0.1 mg/ml) in HCT116 cells, and the expression of p53 was detected by Western blotting. GAPDH was employed as an internal control. **J** CircHAS2 modulated the levels of p53 ubiquitination through the interaction with USP10. HCT116 cells were transfected with the indicated constructs and subjected to MG132 (50 mM) for 4 hours before harvest to evaluate the ubiquitination levels of p53. **K** Representative Western blotting images with indicated treatments in HCT116 cells for Flag, p53, and p21 proteins. GAPDH was used as an internal control. Statistical significance in two-group experiments was assessed using a two-sided Student's t-test. Data are represented as mean ± SD; *, $P < 0.05$; **, $P < 0.01$; ***, $P < 0.001$; ns, no significance.

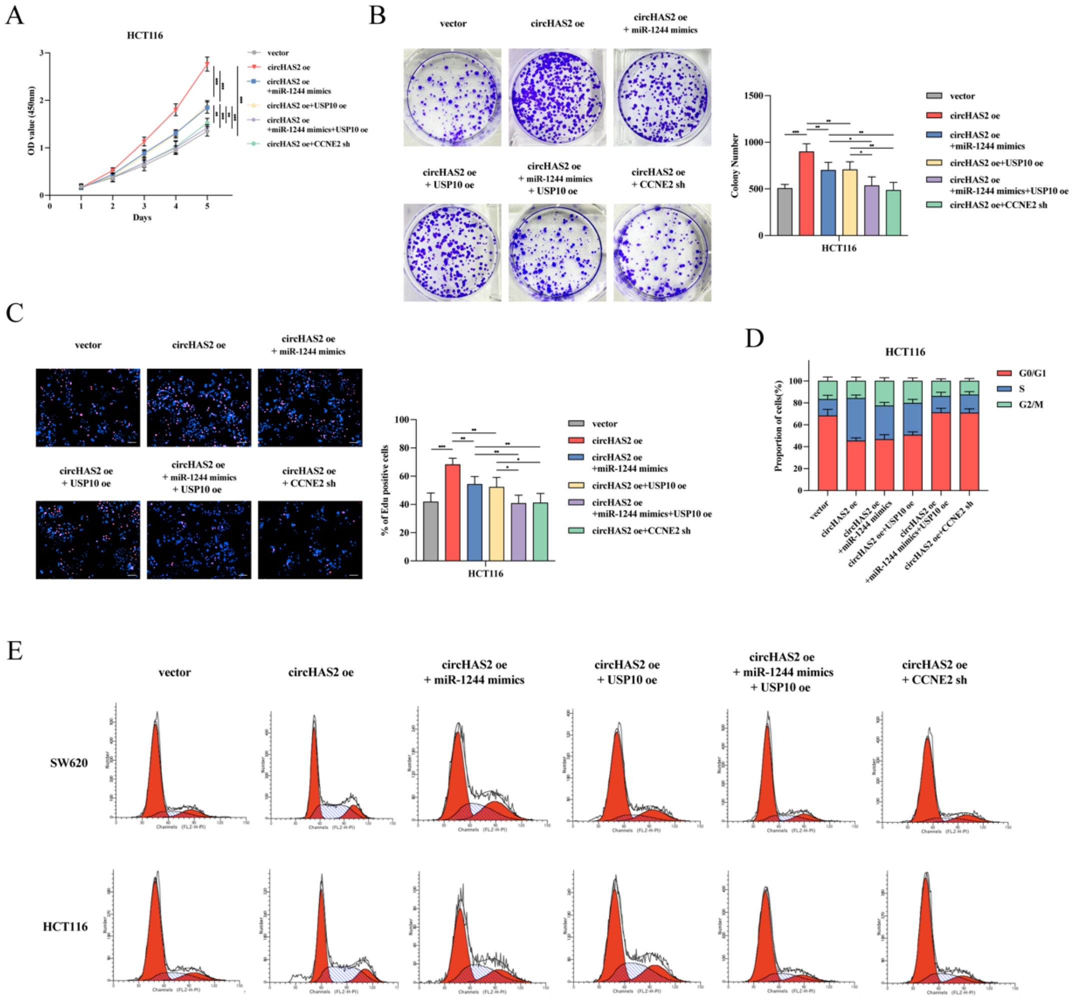

Figure S7 The activation of bidirectional downstream pathways is initiated by circHAS2 to facilitate CRC progression. A The viabilities of HCT116 cells following indicated treatments were detected by CCK-8 assays. **B** Quantitative results and representative images of cell proliferation were conducted by colony formation assay in HCT116 cells following indicated treatments. **C** Quantitative results and representative images of cell proliferation were evaluated by EdU incorporation in HCT116 cells following indicated treatments (scale bar: 50 μm). **D** The percentage of cells in the G1, S, and G2 phases of the entire cell population was determined by flow cytometry following indicated treatments in HCT116 cells. **E** Representative images of the flow cytometry analysis with indicated treatment, illustrating the percentage of cells

in the G1, S, and G2/M phases of the cell cycle relative to the total cell population. Statistical significance in two-group experiments was assessed using a two-sided Student's t-test. Data are represented as mean ± SD; *, $P < 0.05$; **, $P < 0.01$; ***, $P < 0.001$; ns, no significance.

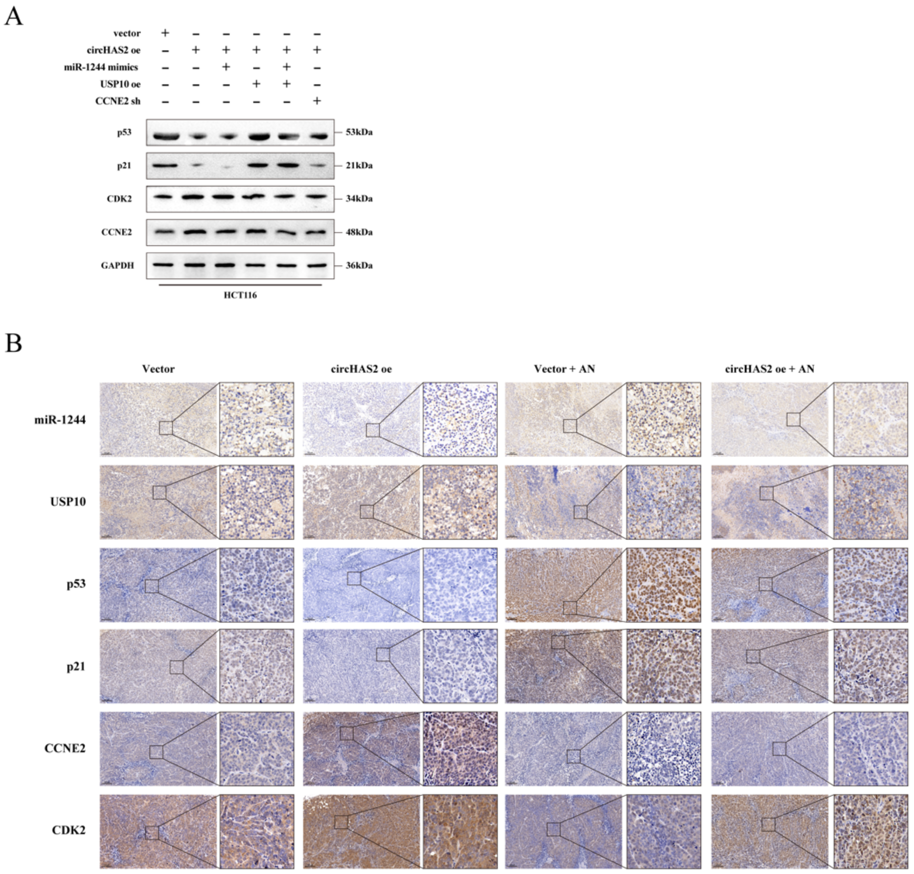

Figure S8 CircHAS2 initiates the activation of bidirectional downstream pathways in CRC. A Representative Western blotting images with indicated treatments for p53, p21, CDK2, and CCNE2 proteins in HCT116 cells. GAPDH was used as an internal control. **B** Representative images of HE staining, ISH staining (miR-1244), and IHC staining (USP10, p53, p21, CCNE2, and CDK2) assay of tumors of indicated treatments (scale bar: 100 μm). Details were magnified at a 5× scale. Data are represented as mean ± SD.

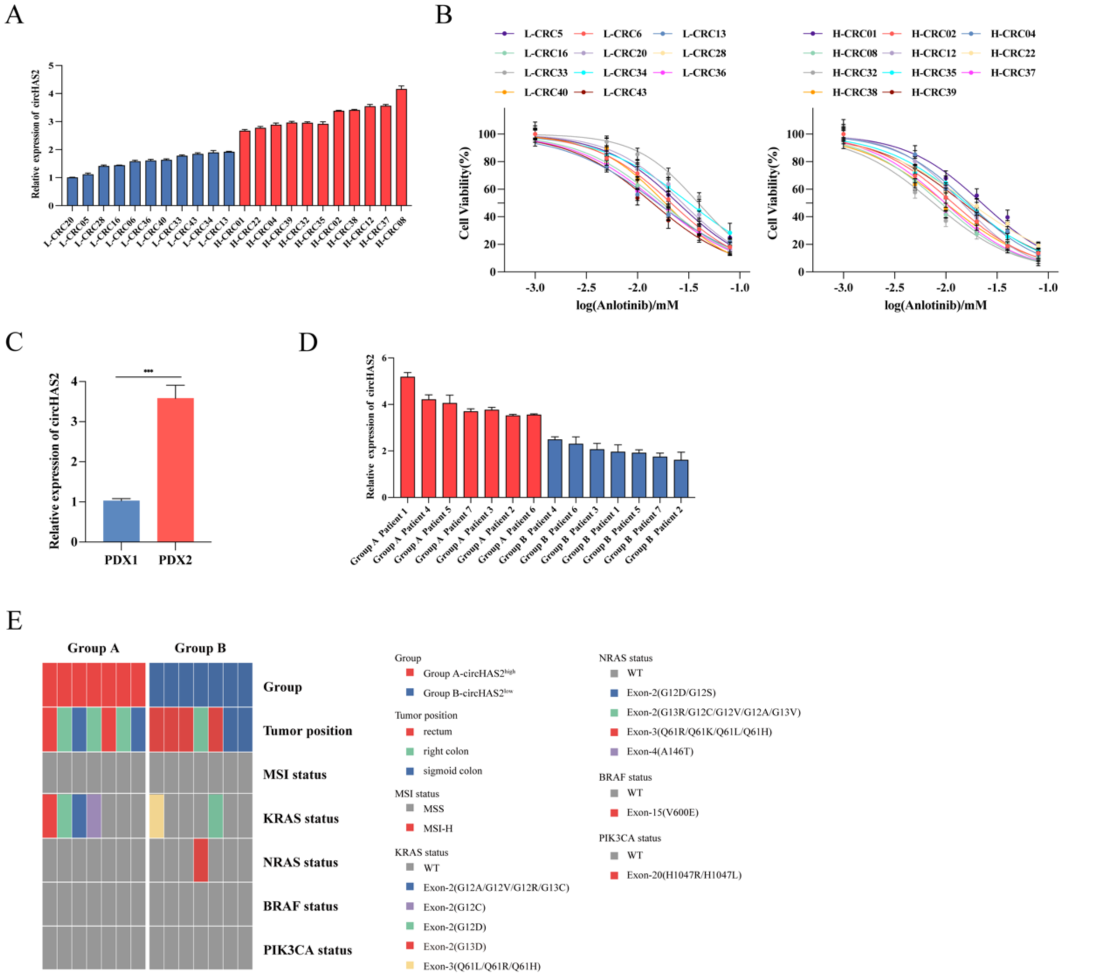

Figure S9 Expression and prognostic effect of circHAS2 in patients with CRC. A The relative expression of circHAS2 was evaluated in 22 PDOs. A score of 2 or less was considered indicative of low expression, while a score exceeding 2 was classified as high expression. **B** Dose-effect curves of organoids treated with the indicated concentrations of anlotinib. **C** The relative expression levels of circHAS2 in the 2 PDX models. **D** The relative expression of circHAS2 was evaluated in 14 patients by qPCR. A score of 3 or less was considered indicative of low expression, while a score exceeding 3 was classified as high expression. **E** Patients in Group A and Group B exhibit variations in clinical information and genetic mutations. Specifically, distinctions in the nature of colorectal cancer are observed based on RAS, BRAF, MSI

status, and tumor location. Statistical significance in two-group experiments was assessed using a two-sided Student's t-test. Data are represented as mean ± SD; *, P < 0.05; **, P < 0.01; ***, P < 0.001; ns, no significance.
