# Supplementary material for: CircHAS2 activates CCNE2 to promote cell proliferation and sensitizes the response of colorectal cancer to anlotinib
Source: Mol Cancer. 2024 Mar 21;23:59. doi: 10.1186/s12943-024-01971-7 (PMC10956180; doi:10.1186/s12943-024-01971-7)
Supplement: Supplementary file 2 — Supplementary Material 2 [file 12943_2024_1971_MOESM2_ESM.pdf]

Table S1 Correlations between circHAS2 expression and clinical characteristics

Clinicopathologic parameters	Case (n = 70)	circHAS2 expression		P value
		Low	High	
Total	70	33	37	
Gender				
Male	41	20	21	0.811
Female	29	13	16	
Age				0.815
≥ 60	40	19	21	
< 60	30	14	18	
Pathological stage				<0.0001*
I-II	29	22	7	
III-IV	41	11	30	
T stage				0.0396*
T1-2	21	14	7	
T3-4	49	19	30	
Lymph node metastasis				0.0001*
N0	32	24	8	
N1	24	6	18	
N2	14	3	11	
Distant metastasis				0.0740
M0	46	26	20	
M1	24	9	17	
TP53 status				
TP53 wildtype	31	18	13	0.1027
TP53 mutation	39	15	24	

*$p < 0.05$ (chi-square test).
