# Supplementary material for: CircHAS2 activates CCNE2 to promote cell proliferation and sensitizes the response of colorectal cancer to anlotinib
Source: Mol Cancer. 2024 Mar 21;23:59. doi: 10.1186/s12943-024-01971-7 (PMC10956180; doi:10.1186/s12943-024-01971-7)
Supplement: Supplementary file 3 — Supplementary Material 3 [file 12943_2024_1971_MOESM3_ESM.pdf]

Table S2 **Univariate and multivariate analysis of clinic pathological factors for overall survival**

Variables	Univariate Analysis HR (95% CI)	P value	Multivariate Analysis HR (95% CI)	P value
circHAS2 (low vs. high)	0.58(0.35-0.95)	0.03*		
Age (≥ 60 vs. < 60)	0.72(0.44-1.15)	0.17		
Gender (male vs. female)	1.04(0.64-1.69)	0.87		
Pathological stage (I-II vs. III-IV)	0.40(0.22-0.70)	<0.001*		
T stage (T1-2 vs. T3-4)	0.49(0.26-0.92)	0.03*		
Lymph node metastasis (N0 vs. N1-2)	0.38(0.22-0.65)	<0.001*		
Distant metastasis (M0 vs. M1)	0.49(0.31-0.80)	<0.001*	0.47(0.25-0.90)	0.02

*$p < 0.05$ (chi-square test).
