# Supplementary material for: CircHAS2 activates CCNE2 to promote cell proliferation and sensitizes the response of colorectal cancer to anlotinib
Source: Mol Cancer. 2024 Mar 21;23:59. doi: 10.1186/s12943-024-01971-7 (PMC10956180; doi:10.1186/s12943-024-01971-7)
Supplement: Supplementary file 4 — Supplementary Material 4 [file 12943_2024_1971_MOESM4_ESM.pdf]

S.Table3 Proteins pulled down by circHAS2

prot_hit_nu	prot_acc	prot_score	prot_mass	prot_matche	prot_matche	prot_sequer	prot_sequer	prot_cover	prot_pi	emPAI
1	sp|P60709|A	13049	42052	367	367	28	28	66.9	5.29	170.2
2	sp|P68032|A	4022	42334	169	169	21	21	37.9	5.23	38.92
3	sp|O75369|F	3735	280157	91	91	66	66	36.2	5.47	1.83
4	sp|O43707|A	3538	105245	104	104	49	49	55.5	5.27	10.59
5	sp|P21333|F	2688	283301	68	68	54	54	30.1	5.7	1.15
6	sp|Q9BYX7|A	2393	42331	55	55	5	5	13.1	5.91	1.12
7	sp|P09327|V	2368	93093	66	66	31	31	47.5	5.99	4.08
8	sp|P12814|A	2110	103563	52	52	34	34	44.4	5.25	3.45
9	sp|Q562R1|A	1982	42318	84	84	9	9	26.9	5.39	3.5
10	sp|Q13813|S	1705	285163	40	40	35	35	19.4	5.22	0.58
11	sp|P13797|P	1216	71279	31	31	22	22	34.8	5.41	2.69
12	sp|P06396|G	1194	86043	24	24	13	13	28.3	5.9	1.03
13	sp|P61160|A	1098	45017	22	22	11	11	32	6.3	2.33
14	sp|O15144|A	932	34426	34	34	13	13	42.3	6.84	6.57
15	sp|O00515|L	908	57154	24	24	12	12	30.2	9.67	2.24
16	sp|Q9BQE3|T	896	50548	25	25	16	16	42.1	4.96	3.02
17	sp|O15020|S	829	272526	18	18	18	18	10.7	5.79	0.24
18	sp|P07437|T	788	50095	22	22	15	15	41.4	4.78	2.58
19	sp|P09211|G	787	23569	12	12	8	8	56.2	5.43	3.93
20	sp|P07355|A	770	38808	19	19	13	13	38.6	7.57	3.73
21	sp|P61158|A	759	47797	27	27	14	14	36.6	5.61	2.79
22	sp|Q14247|S	750	61720	16	16	13	13	30.7	5.24	1.29
23	sp|P68371|T	713	50255	19	19	13	13	34.6	4.79	2.14
24	sp|Q9UID3|V	697	86901	14	14	12	12	19.4	6.06	0.68
25	sp|P52272|H	692	77749	15	15	12	12	22.1	8.84	0.78
26	sp|P22626|R	677	37464	17	17	12	12	39.7	8.97	3.21
27	sp|P09651|R	664	38837	18	18	12	12	36.3	9.17	3.36
28	sp|P49327|F	638	275877	21	21	20	20	11.4	6.01	0.28
29	sp|P46940|I	615	189761	16	16	14	14	11.8	6.08	0.31
30	sp|Q00610|C	615	193260	15	15	15	15	12.7	5.48	0.29
31	sp|P05787|K	606	53671	19	19	16	16	36.9	5.52	2.1
32	sp|P08727|K	595	44079	14	14	13	13	45.3	5.04	1.75
33	sp|P13796|P	593	70814	18	18	15	15	29	5.29	1.16
34	sp|Q8N1B4|	575	82512	13	13	11	11	23.9	5.7	0.6
35	sp|Q96C19|E	533	26794	18	18	12	12	43.3	5.15	6.37
36	sp|P60842|I	532	46353	10	10	9	9	28.3	5.32	0.99
37	sp|P14618|K	521	58470	13	13	10	10	25	7.96	1.04
38	sp|P68366|T	514	50634	16	16	11	11	30.1	4.95	1.42
39	sp|P06576|A	513	56525	12	12	12	12	34.6	5.26	0.97
40	sp|Q8IVT2|M	512	75482	14	14	12	12	25.8	6.36	0.81
41	sp|Q9H267|V	511	71225	16	16	14	14	21.4	6.29	1.06
42	sp|O60506|H	503	69788	10	10	9	9	19.9	8.68	0.58
43	sp|P55072|T	498	89950	13	13	11	11	17.2	5.14	0.59
44	sp|Q9NYL9|	483	39741	11	11	8	8	34.9	5.08	1.22
45	sp|P04264|K	465	66170	7	7	6	6	18.9	8.15	0.4
46	sp|Q9H9C1|S	449	57198	11	11	10	10	24.7	6.94	0.85
47	sp|O14950|M	447	19824	9	9	6	6	44.2	4.71	3.09
48	sp|P78371|T	443	57794	7	7	6	6	18.3	6.01	0.47
49	sp|Q92616|G	441	294967	8	8	7	7	4.1	7.29	0.09
50	sp|P30153|2	432	66065	11	11	11	11	25.6	5	0.71
51	sp|P11940|P	426	70854	11	11	10	10	18.6	9.52	0.65
52	sp|P49368|T	412	61066	8	8	7	7	18.9	6.1	0.52
53	sp|O94832|M	409	116927	11	11	8	8	11.2	9.44	0.35
54	sp|P52597|H	403	45985	9	9	7	7	25.5	5.38	0.87
55	sp|P31943|H	403	49484	9	9	5	5	15.8	5.89	0.67
56	sp|Q5VIR6|V	384	95200	12	12	10	10	14.7	6.29	0.5
57	sp|O00425|I	384	64008	8	8	8	8	18.7	8.99	0.49
58	sp|Q07666|K	383	48311	13	13	7	7	24.2	8.73	1.21

59	sp|P23528|C	381	18719	7	7	5	5	42.2	8.22	2.18
60	sp|P05783|K	381	48029	7	7	6	6	21.6	5.34	0.59
61	sp|P22314|U	366	118858	10	10	8	8	12.3	5.49	0.31
62	sp|P52907|C	363	33073	8	8	6	6	39.9	5.45	1.15
63	sp|P61978|H	357	51230	12	12	11	11	29.4	5.39	1.11
64	sp|P62136|P	354	38229	11	11	8	8	24.2	5.94	1.5
65	sp|P67809|Y	347	35903	8	8	6	6	38.3	9.87	1.03
66	sp|P26599|P	340	57357	5	5	3	3	9.6	9.22	0.32
67	sp|Q96FF7|N	335	24128	6	6	5	5	37.4	10.92	1.18
68	sp|O75533|S	332	146479	9	9	8	8	7.1	6.65	0.22
69	sp|Q12929|E	318	92167	5	5	5	5	10.5	7.1	0.19
70	sp|Q96JG6|V	309	111959	9	9	8	8	11.3	5.85	0.3
71	sp|P10809|C	306	61187	7	7	7	7	19.7	5.7	0.44
72	sp|P30101|P	306	57146	11	11	9	9	27.5	5.98	0.85
73	sp|P11142|H	305	71082	7	7	5	5	11.3	5.37	0.37
74	sp|Q9NZI8|I	302	63783	5	5	5	5	11.8	9.26	0.29
75	sp|P60660|N	301	17090	7	7	5	5	34.4	4.56	2.54
76	sp|Q08211|D	301	142181	9	9	9	9	10	6.41	0.23
77	sp|P36578|R	299	47953	10	10	7	7	21.1	11.07	0.94
78	sp|P12956|X	297	70084	12	12	10	10	22.2	6.23	0.73
79	sp|P05388|R	297	34423	5	5	4	4	23	5.71	0.58
80	sp|P38159|R	294	42306	9	9	9	9	20.7	10.06	0.97
81	sp|P62805|H	292	11360	8	8	6	6	51.5	11.36	7.38
82	sp|P55060|X	289	111145	11	11	10	10	11.9	5.51	0.34
83	sp|Q7L576|C	285	146742	10	10	9	9	8.9	6.46	0.25
84	sp|P04844|R	279	69355	5	5	4	4	13.3	5.44	0.26
85	sp|Q14651|P	278	70608	11	11	9	9	21.1	5.28	0.58
86	sp|O43390|H	277	71184	7	7	7	7	12.3	8.23	0.37
87	sp|Q01082|S	276	275237	6	6	6	6	3.8	5.39	0.07
88	sp|Q92747|A	275	42113	7	7	4	4	14.3	8.46	0.7
89	sp|P13010|X	275	83222	10	10	9	9	19.9	5.55	0.47
90	sp|P47755|C	273	33157	6	6	4	4	21	5.57	0.61
91	sp|P04075|A	272	39851	7	7	5	5	19.8	8.3	0.75
92	sp|O94875|S	268	125171	8	8	5	5	7.5	8.56	0.2
93	sp|P62937|P	267	18229	9	9	6	6	35.2	7.68	3.62
94	sp|Q96JY6|P	265	37835	6	6	5	5	26.7	9	0.65
95	sp|P25705|A	262	59828	7	7	7	7	15.9	9.16	0.45
96	sp|Q12905|II	259	43263	5	5	5	5	18.5	5.19	0.44
97	sp|P51991|R	255	39799	7	7	5	5	16.4	9.1	0.75
98	sp|P04406|G	254	36201	6	6	5	5	24.8	8.57	0.69
99	sp|P50990|T	250	60153	4	4	4	4	9.3	5.42	0.24
100	sp|P07737|P	248	15216	6	6	6	6	40.7	8.44	2.35
101	sp|Q92841|D	245	80906	8	8	8	8	13.9	8.53	0.37
102	sp|P37802|T	244	22548	6	6	6	6	40.7	8.41	1.3
103	sp|Q5VTE0|E	244	50495	7	7	5	5	14.5	9.15	0.56
104	sp|P06733|E	243	47481	4	4	4	4	17.7	7.01	0.31
105	sp|Q14697|G	240	107263	5	5	5	5	7.2	5.74	0.16
106	sp|O15511|A	239	16367	6	6	4	4	33.1	5.47	1.57
107	sp|O43175|S	235	57356	6	6	6	6	14.6	6.29	0.4
108	sp|P35232|P	235	29843	5	5	5	5	23.9	5.57	0.7
109	sp|P00338|L	234	36950	8	8	7	7	18.7	8.44	0.99
110	sp|O15143|A	233	41722	7	7	5	5	16.7	8.69	0.7
111	sp|P27635|R	232	25044	5	5	4	4	14.5	10.11	0.87
112	sp|Q9BQI0|A	231	17114	4	4	3	3	28	6.63	1.06
113	sp|P13645|K	229	59020	7	7	6	6	12.3	5.13	0.46
114	sp|O00571|D	228	73597	7	7	7	7	14.8	6.73	0.36
115	sp|Q12906|II	226	95678	6	6	6	6	10.3	8.86	0.22
116	sp|P04792|H	224	22826	5	5	4	4	24.4	5.98	0.98
117	sp|P47756|C	223	31616	4	4	4	4	17.3	5.36	0.49
118	sp|P62979|R	217	18296	7	7	4	4	30.1	9.68	1.76

119 sp|P63104|1	213	27899	3	3	3	3	17.1	4.73	0.4
120 sp|P35222|C	211	86069	6	6	6	6	10	5.53	0.25
121 sp|P05387|R	205	11658	4	4	3	3	53.9	4.42	1.81
122 sp|Q14974|II	203	98420	5	5	5	5	7.5	4.68	0.18
123 sp|P35637|F	199	53622	3	3	3	3	7.6	9.4	0.2
124 sp|P18621|R	197	21611	5	5	4	4	20.1	10.18	1.06
125 sp|Q9Y2D5|	195	95002	3	3	3	3	5.9	5.04	0.11
126 sp|P50395|G	190	51087	4	4	3	3	9.7	6.11	0.28
127 sp|P0DP23|C	189	16827	4	4	3	3	34.9	4.09	1.08
128 sp|P17844|D	189	69618	7	7	6	6	11.6	9.06	0.38
129 sp|Q86V81|T	187	26872	5	5	3	3	18.3	11.15	0.8
130 sp|Q96F07|C	185	150298	7	7	6	6	5.7	7.03	0.16
131 sp|Q9Y490|T	184	271766	3	3	3	3	1.6	5.77	0.04
132 sp|Q13200|P	181	100877	3	3	2	2	2.8	5.08	0.1
133 sp|Q15393|S	180	136575	5	5	5	5	5.9	5.13	0.13
134 sp|P08195|4	179	68180	3	3	3	3	4.6	4.89	0.15
135 sp|P46777|R	177	34569	4	4	2	2	8.8	9.73	0.44
136 sp|Q9NZQ3	176	79651	4	4	4	4	6.6	5.94	0.18
137 sp|O00487|P	175	34726	2	2	1	1	7.1	6.06	0.2
138 sp|P26641|E	171	50429	5	5	5	5	14.4	6.25	0.37
139 sp|Q15233|N	171	54311	4	4	3	3	9.8	9.01	0.27
140 sp|Q96JP2|M	169	168066	4	4	4	4	3.9	8.75	0.08
141 sp|P10412|H	169	21852	4	4	3	3	17.4	11.03	0.77
142 sp|P00558|P	169	44985	5	5	4	4	18.7	8.3	0.43
143 sp|A0AV96|F	166	64514	2	2	2	2	6.7	7.56	0.1
144 sp|P60903|S	165	11310	4	4	2	2	35.1	6.82	1.89
145 sp|Q13310|P	165	71080	3	3	3	3	8.2	9.31	0.15
146 sp|P23396|R	165	26842	7	7	7	7	38.3	9.68	1.27
147 sp|Q96PK6|F	163	69620	4	4	4	4	7.9	9.68	0.2
148 sp|O60716|C	161	108674	6	6	4	4	8.2	5.86	0.19
149 sp|P62277|R	160	17212	7	7	6	6	31.1	10.53	2.51
150 sp|P35908|K	160	65678	3	3	3	3	5.6	8.07	0.16
151 sp|Q14103|H	159	38581	4	4	4	4	11.5	7.62	0.39
152 sp|Q9P1U1|	158	48090	5	5	5	5	15.3	5.61	0.39
153 sp|Q15717|E	156	36240	4	4	4	4	15.3	9.23	0.42
154 sp|P31942|H	154	36960	4	4	4	4	21.4	6.37	0.41
155 sp|P62424|R	154	30148	5	5	5	5	18.4	10.61	0.69
156 sp|P05141|A	152	33059	5	5	4	4	15.4	9.71	0.61
157 sp|Q9Y2A7|I	152	130018	4	4	4	4	5.2	6.18	0.1
158 sp|Q92552|F	151	47924	2	2	1	1	5.8	5.83	0.14
159 sp|P12236|A	148	33073	5	5	4	4	15.4	9.76	0.61
160 sp|P30050|R	145	17979	3	3	3	3	24.2	9.48	0.68
161 sp|P16401|H	143	22566	5	5	4	4	17.7	10.91	1
162 sp|P42766|R	143	14543	4	4	2	2	22	11.04	1.32
163 sp|P62847|R	142	15413	3	3	3	3	29.3	10.79	0.82
164 sp|Q04837|S	142	17249	3	3	3	3	26.4	9.59	0.71
165 sp|P61247|R	140	30154	3	3	3	3	15.9	9.75	0.37
166 sp|P11586|C	140	102180	4	4	4	4	5.3	6.89	0.13
167 sp|Q9Y6M1|	139	66195	4	4	4	4	8.5	8.48	0.21
168 sp|Q9H299|S	137	10488	3	3	2	2	31.2	4.82	1.36
169 sp|P07237|P	137	57480	4	4	4	4	9.1	4.76	0.25
170 sp|O15145|A	137	20761	3	3	3	3	16.3	8.78	0.57
171 sp|P49588|S	136	107484	2	2	2	2	2.8	5.34	0.06
172 sp|Q5QNW6	134	13912	3	3	2	2	19	10.31	0.55
173 sp|P52292|IN	134	58168	2	2	2	2	6.6	5.25	0.12
174 sp|P15531|N	133	17309	3	3	2	2	19.1	5.83	0.71
175 sp|P04843|R	133	68641	4	4	4	4	10	5.96	0.21
176 sp|P38919|IF	132	47126	4	4	4	4	14.4	6.3	0.31
177 sp|P60866|R	132	13478	4	4	3	3	22.7	9.95	1.48
178 sp|P07195|L	132	36900	5	5	5	5	18	5.71	0.54

179	sp|P59998|A	131	19768	5	5	4	4	31	8.53	1.2
180	sp|P62306|R	131	9776	2	2	1	1	15.1	4.7	0.36
181	sp|P27348|1	131	28032	2	2	2	2	9.8	4.68	0.25
182	sp|P62269|R	130	17708	3	3	3	3	18.4	10.99	0.69
183	sp|P53618|C	130	108214	3	3	3	3	4.6	5.72	0.09
184	sp|O00303|E	129	37654	2	2	2	2	7.6	5.24	0.18
185	sp|P36957|C	128	49067	3	3	3	3	7.5	9.11	0.22
186	sp|P50991|T	128	58401	3	3	2	2	6.1	7.96	0.18
187	sp|Q9UJU6|D	128	48463	3	3	3	3	9.5	5.02	0.22
188	sp|O75390|C	126	51908	3	3	3	3	7.9	8.45	0.2
189	sp|P46778|R	123	18610	3	3	2	2	16.3	10.49	0.65
190	sp|Q92804|R	123	62021	3	3	2	2	5.2	8.04	0.17
191	sp|P39023|R	122	46365	3	3	3	3	14.1	10.19	0.23
192	sp|O95684|C	121	43153	1	1	1	1	5.3	4.67	0.08
193	sp|P62081|R	119	22113	3	3	2	2	17.5	10.09	0.53
194	sp|Q9BPX5|A	117	16931	4	4	3	3	28.8	6.15	1.07
195	sp|Q9Y2T7|Y	117	38552	4	4	3	3	14.3	10.8	0.39
196	sp|Q9Y446|P	117	87485	5	5	5	5	8.5	9.39	0.2
197	sp|P15559|N	117	30905	6	6	4	4	14.6	8.91	0.85
198	sp|P14324|F	115	48758	2	2	1	1	4.3	5.83	0.14
199	sp|P26373|R	115	24304	4	4	4	4	17.5	11.65	0.67
200	sp|Q9BXK1|I	113	25871	1	1	1	1	10.3	9.95	0.13
201	sp|Q7KZF4|S	113	102618	3	3	3	3	4.9	6.74	0.1
202	sp|P62314|S	113	13273	3	3	2	2	27.7	11.56	0.98
203	sp|P62917|R	112	28235	3	3	2	2	10.9	11.03	0.4
204	sp|P40227|T	112	58444	4	4	4	4	9.8	6.23	0.24
205	sp|Q9UKM9	111	32501	3	3	3	3	21.6	9.2	0.34
206	sp|P26640|S	111	141642	2	2	2	2	2.5	7.53	0.05
207	sp|Q96FW1|	111	31492	2	2	1	1	7	4.85	0.22
208	sp|Q9Y3B4|S	109	14690	2	2	2	2	20.8	9.41	0.52
209	sp|P62249|R	108	16549	3	3	3	3	19.9	10.21	0.75
210	sp|P37108|S	108	14675	1	1	1	1	10.3	10.05	0.23
211	sp|P05386|R	107	11621	2	2	2	2	28.9	4.26	0.68
212	sp|P06239|L	107	58477	2	2	2	2	5.7	5.23	0.12
213	sp|P61981|1	107	28456	3	3	3	3	18.6	4.8	0.39
214	sp|P0DMV8|	106	70294	2	2	2	2	4.5	5.48	0.1
215	sp|O43815|S	106	86535	3	3	3	3	6.8	5.12	0.12
216	sp|P84090|E	106	12422	3	3	2	2	20.2	5.63	1.08
217	sp|P62258|1	106	29326	5	5	4	4	19.6	4.63	0.71
218	sp|P15374|U	105	26337	2	2	2	2	12.6	4.84	0.27
219	sp|P60174|T	104	26938	3	3	3	3	16.9	6.45	0.42
220	sp|Q8NF37|I	104	59741	1	1	1	1	2.8	5.77	0.06
221	sp|P46783|R	104	18886	4	4	3	3	18.8	10.15	0.93
222	sp|P62195|P	103	45768	2	2	2	2	9.9	7.11	0.15
223	sp|P62888|R	102	12947	2	2	2	2	10.4	9.65	0.6
224	sp|Q92900|R	102	125578	2	2	2	2	2.6	6.18	0.05
225	sp|P12268|IN	102	56226	2	2	2	2	6.6	6.44	0.12
226	sp|P31939|P	101	65089	3	3	3	3	6.4	6.27	0.16
227	sp|P62241|R	101	24475	2	2	2	2	11.5	10.32	0.29
228	sp|P62753|R	98	28834	2	2	2	2	10.8	10.85	0.24
229	sp|Q9UHB6|	98	85630	4	4	3	3	5.5	6.41	0.16
230	sp|P29692|E	97	31217	3	3	3	3	12.1	4.9	0.35
231	sp|P53992|S	97	119789	1	1	1	1	1.6	6.71	0.03
232	sp|Q9NQ55|	96	53446	1	1	1	1	3	10.13	0.06
233	sp|Q9UJZ1|S	96	38624	2	2	2	2	9	6.88	0.18
234	sp|Q8IZP0|A	95	55161	4	4	3	3	8.9	6.57	0.26
235	sp|P13693|T	95	19697	2	2	2	2	15.7	4.84	0.37
236	sp|Q9BVQ7|	94	81515	1	1	1	1	2.5	8.43	0.04
237	sp|P58107|E	94	557768	1	1	1	1	0.5	5.45	0.01
238	sp|O95425|S	94	249417	2	2	2	2	1.7	6.55	0.03

239	sp|P48643|T	93	60089	3	3	3	3	7.4	5.45	0.17
240	sp|P32322|P	93	33568	1	1	1	1	5.6	7.18	0.1
241	sp|P24534|E	93	24919	1	1	1	1	6.7	4.5	0.13
242	sp|P17987|T	92	60819	5	5	4	4	9.2	5.8	0.23
243	sp|Q99832|T	91	59842	6	6	4	4	8.1	7.55	0.38
244	sp|Q9Y230|F	91	51296	2	2	2	2	5.6	5.49	0.13
245	sp|O60664|P	91	47217	2	2	2	2	7.6	5.3	0.14
246	sp|Q9Y305|A	90	50269	2	2	2	2	7.3	8.81	0.14
247	sp|P02768|A	89	71317	2	2	1	1	2.5	5.92	0.09
248	sp|Q63ZY3|K	88	91916	1	1	1	1	2.5	5.44	0.04
249	sp|P08133|A	88	76168	1	1	1	1	2.4	5.42	0.04
250	sp|P14866|H	87	64720	2	2	2	2	4.6	8.46	0.1
251	sp|Q15365|P	87	37987	1	1	1	1	5.6	6.66	0.09
252	sp|P0DOX5|	86	49925	8	8	2	2	3.3	8.94	0.21
253	sp|P04632|C	85	28469	1	1	1	1	5.6	5.05	0.12
254	sp|P62750|R	85	17684	3	3	3	3	20.5	10.44	0.69
255	sp|P50454|S	85	46525	2	2	2	2	9.8	8.75	0.15
256	sp|Q8TCD5|I	85	23596	1	1	1	1	9	6.18	0.14
257	sp|P83731|R	85	17882	3	3	3	3	19.1	11.26	0.68
258	sp|Q96ER9|M	85	46011	1	1	1	1	5.4	8.3	0.07
259	sp|P50416|C	85	88995	1	1	1	1	1.8	8.85	0.04
260	sp|O00148|D	84	49611	2	2	2	2	5.6	5.46	0.14
261	sp|Q9BUJ2|H	84	96250	2	2	2	2	3.4	6.49	0.07
262	sp|Q01518|C	83	52325	1	1	1	1	4.8	8.24	0.06
263	sp|Q99871|H	83	41550	1	1	1	1	4.3	4.73	0.08
264	sp|P12429|A	83	36524	2	2	2	2	7.7	5.63	0.19
265	sp|Q13418|II	83	51899	2	2	2	2	4.4	8.3	0.13
266	sp|Q9BY77|F	82	46289	1	1	1	1	3.3	10	0.07
267	sp|P49006|M	81	19574	1	1	1	1	7.7	4.68	0.17
268	sp|Q9UQ80|	81	44101	2	2	2	2	7.9	6.13	0.16
269	sp|P13639|E	80	96246	4	4	4	4	5.1	6.41	0.14
270	sp|Q9NUQ6	80	62204	1	1	1	1	3	9.67	0.05
271	sp|Q8WWM	80	113589	1	1	1	1	2	8.7	0.03
272	sp|O95433|A	80	38421	1	1	1	1	6.5	5.41	0.09
273	sp|Q5T1J5|C	79	15708	1	1	1	1	18.5	9.95	0.22
274	sp|Q53HC9|I	79	44032	3	3	3	3	13.7	4.87	0.24
275	sp|Q99460|P	78	106795	1	1	1	1	1.3	5.25	0.03
276	sp|O14980|X	78	124447	3	3	3	3	3.2	5.71	0.08
277	sp|P62263|R	78	16434	1	1	1	1	13.9	10.07	0.21
278	sp|P62495|E	78	49228	1	1	1	1	3	5.51	0.07
279	sp|P27708|P	78	245167	2	2	2	2	0.9	6.02	0.03
280	sp|P0C0S8|H	78	14083	2	2	2	2	21.5	10.9	0.54
281	sp|Q3LXA3|T	77	59252	2	2	2	2	7.1	7.12	0.11
282	sp|Q14166|T	76	75154	2	2	2	2	5	5.33	0.09
283	sp|P62333|P	76	44430	2	2	2	2	7.5	7.1	0.15
284	sp|Q9Y2L1|F	76	110017	1	1	1	1	1.4	6.69	0.03
285	sp|P56192|S	75	102249	2	2	2	2	2.3	5.82	0.07
286	sp|Q68CZ2|T	75	156366	2	2	2	2	2.1	6.34	0.04
287	sp|P17980|P	74	49458	1	1	1	1	3.6	5.13	0.07
288	sp|Q9Y265|F	74	50538	1	1	1	1	3.5	6.02	0.07
289	sp|P69905|H	73	15305	4	4	3	3	23.2	8.72	1.23
290	sp|P63173|R	73	8270	2	2	1	1	17.1	10.1	1.02
291	sp|Q07020|R	73	21735	2	2	2	2	13.8	11.73	0.33
292	sp|Q7L5N1|C	72	36482	1	1	1	1	5.5	5.47	0.09
293	sp|Q13283|G	72	52189	1	1	1	1	3.6	5.36	0.06
294	sp|Q99439|C	71	34074	2	2	2	2	9.1	6.95	0.2
295	sp|P53396|A	71	121674	2	2	2	2	2.6	6.95	0.05
296	sp|O00299|C	71	27248	2	2	2	2	11.2	5.09	0.26
297	sp|P62829|R	71	14970	1	1	1	1	12.9	10.51	0.23
298	sp|Q9BXW7	70	46748	1	1	1	1	3.1	8.38	0.07

299	sp|Q96IJ6|GI	70	46604	1	1	1	1	3.3	6.73	0.07
300	sp|Q99598|T	70	33206	1	1	1	1	6.6	6.1	0.1
301	sp|P34897|G	69	56414	2	2	2	2	4	8.76	0.12
302	sp|P46776|R	69	16665	1	1	1	1	7.4	11	0.2
303	sp|P50914|R	69	23531	1	1	1	1	5.6	10.94	0.14
304	sp|P37268|F	68	48597	1	1	1	1	4.3	6.1	0.07
305	sp|P57088|T	68	28302	2	2	2	2	8.1	9.75	0.25
306	sp|P45880|V	68	32060	1	1	1	1	3.4	7.49	0.1
307	sp|Q9BSJ8|E	68	123293	1	1	1	1	1.2	5.57	0.03
308	sp|Q9Y678|C	68	98967	2	2	2	2	3.1	5.32	0.07
309	sp|P50552|V	68	39976	1	1	1	1	4.2	9.05	0.08
310	sp|Q14444|C	67	78489	1	1	1	1	2.7	5.14	0.04
311	sp|A8MWD9	66	8595	1	1	1	1	17.1	8.93	0.41
312	sp|P62854|R	66	13292	1	1	1	1	13	11.01	0.26
313	sp|O75436|V	66	38260	1	1	1	1	5.2	6.13	0.09
314	sp|A5YKK6|C	66	269106	4	4	4	4	1.9	6.65	0.05
315	sp|Q7KZI7|M	66	88255	1	1	1	1	2.5	9.73	0.04
316	sp|Q13425|S	66	58369	1	1	1	1	1.7	9.03	0.06
317	sp|Q96EY1|D	66	53083	1	1	1	1	3.5	9.37	0.06
318	sp|P39656|C	66	50940	2	2	2	2	4.8	6.09	0.13
319	sp|P09960|L	66	69868	2	2	2	2	4.1	5.8	0.1
320	sp|Q15758|A	66	57018	2	2	2	2	5.7	5.34	0.12
321	sp|P60953|C	66	21587	1	1	1	1	8.9	6.15	0.16
322	sp|Q07021|C	66	31742	1	1	1	1	5	4.74	0.1
323	sp|P22234|P	66	47790	1	1	1	1	3.3	6.95	0.07
324	sp|Q13045|F	65	146142	1	1	1	1	0.8	5.75	0.02
325	sp|Q9UMS4|	65	55603	1	1	1	1	3.4	6.14	0.06
326	sp|Q8NEV1|	65	45305	1	1	1	1	4.6	8.54	0.07
327	sp|O75128|C	65	136446	1	1	1	1	1.3	7.66	0.02
328	sp|Q01844|E	65	68721	1	1	1	1	2.1	9.37	0.05
329	sp|P42704|L	64	159003	3	3	3	3	2.2	5.81	0.06
330	sp|Q00653|N	64	97373	1	1	1	1	1.6	5.84	0.03
331	sp|Q9Y3U8|I	63	12303	2	2	2	2	19	11.59	0.63
332	sp|P10155|R	63	61372	2	2	2	2	4.5	8.27	0.11
333	sp|P61923|C	63	20242	1	1	1	1	10.7	4.69	0.17
334	sp|P19367|H	63	103561	1	1	1	1	1.3	6.36	0.03
335	sp|Q15459|S	62	88888	1	1	1	1	2	5.15	0.04
336	sp|P08758|A	62	35971	2	2	2	2	6.3	4.94	0.19
337	sp|Q13263|T	62	90261	2	2	2	2	3.7	5.52	0.07
338	sp|P04004|V	62	55069	1	1	1	1	3.1	5.55	0.06
339	sp|Q14671|F	61	127079	1	1	1	1	1.1	6.35	0.03
340	sp|Q13015|A	61	10055	1	1	1	1	15.6	4.4	0.34
341	sp|Q6NYC8|	61	68187	1	1	1	1	3.8	5.38	0.05
342	sp|Q9UIL1|S	61	18148	1	1	1	1	11.3	9	0.19
343	sp|P00491|P	60	32325	1	1	1	1	3.5	6.45	0.1
344	sp|Q9BRA2|	60	14217	1	1	1	1	11.4	5.4	0.24
345	sp|Q9Y6E2|E	60	48360	2	2	2	2	6.7	6.26	0.14
346	sp|P28066|P	60	26565	1	1	1	1	5	4.74	0.13
347	sp|P01024|C	60	188569	1	1	1	1	1.2	6.02	0.02
348	sp|Q96QK1|	59	92447	1	1	1	1	1.8	5.32	0.04
349	sp|P49755|T	59	25131	1	1	1	1	5	6.97	0.13
350	sp|P62318|S	59	14021	2	2	2	2	15.1	10.33	0.55
351	sp|P26196|D	59	54781	1	1	1	1	5	8.85	0.06
352	sp|O75340|P	59	21912	1	1	1	1	5.8	5.16	0.15
353	sp|P60900|P	59	27838	1	1	1	1	7.3	6.34	0.12
354	sp|P11413|G	58	59675	2	2	2	2	3.5	6.39	0.11
355	sp|P15880|R	58	31590	4	4	3	3	11.6	10.25	0.49
356	sp|Q13887|K	58	51273	2	2	1	1	5.9	8.86	0.13
357	sp|P31146|C	58	51678	1	1	1	1	3.5	6.25	0.06
358	sp|Q00526|C	58	35138	1	1	1	1	3.6	8.86	0.09

359	sp|Q8WXE9|	58	101843	1	1	1	1	1	5.2	0.03
360	sp|P23246|S	58	76216	2	2	2	2	5.7	9.45	0.09
361	sp|P52926|H	58	11825	1	1	1	1	20.2	10.63	0.29
362	sp|Q08J23|N	58	87214	1	1	1	1	1.4	6.33	0.04
363	sp|Q16643|D	58	71842	2	2	1	1	2.5	4.41	0.09
364	sp|Q92615|L	58	80902	1	1	1	1	2.3	6.48	0.04
365	sp|Q99959|P	57	97868	1	1	1	1	1.2	9.39	0.03
366	sp|O00232|P	57	53270	1	1	1	1	2.9	7.53	0.06
367	sp|Q9NYB9|	57	55686	2	2	2	2	5.7	5.82	0.12
368	sp|P33991|M	57	97068	1	1	1	1	2.8	6.28	0.03
369	sp|P48444|C	56	57630	1	1	1	1	2.3	5.89	0.06
370	sp|P38646|G	56	73920	1	1	1	1	1.8	5.87	0.04
371	sp|P18124|R	56	29264	2	2	1	1	7.7	10.66	0.24
372	sp|Q8WXE0|	56	127276	1	1	1	1	2	6.63	0.03
373	sp|Q99729|R	55	36316	2	2	2	2	8.7	8.22	0.19
374	sp|O00151|P	55	36505	2	2	2	2	9.4	6.56	0.19
375	sp|O60488|A	55	80220	1	1	1	1	2.5	8.66	0.04
376	sp|Q27J81|IN	54	136851	1	1	1	1	1.2	5.26	0.02
377	sp|Q9BQ67|	54	49787	1	1	1	1	4.9	4.82	0.07
378	sp|P35268|R	54	14835	1	1	1	1	10.2	9.21	0.23
379	sp|Q8NBS9|	54	48283	2	2	2	2	6.7	5.63	0.14
380	sp|P30740|IL	54	42829	2	2	2	2	6.3	5.9	0.16
381	sp|Q15427|S	53	44414	1	1	1	1	5.7	8.54	0.07
382	sp|P52565|G	53	23250	1	1	1	1	7.8	5.02	0.14
383	sp|Q08AM6|	53	88943	1	1	1	1	2	5.76	0.04
384	sp|Q14694|U	53	87707	1	1	1	1	1.5	5.19	0.04
385	sp|P39019|R	53	16051	3	3	3	3	11.7	10.31	0.77
386	sp|Q9ULV4|	53	53899	1	1	1	1	3.4	6.65	0.06
387	sp|P33240|C	53	61035	1	1	1	1	3.6	6.36	0.05
388	sp|Q8N0Y7|	53	28930	1	1	1	1	5.5	6.19	0.11
389	sp|P82921|R	52	10909	1	1	1	1	13.8	9.96	0.32
390	sp|P05198|IF	52	36374	1	1	1	1	3.8	5.02	0.09
391	sp|Q6IS14|IF	52	16990	1	1	1	1	7.8	4.85	0.2
392	sp|P35527|K	52	62255	2	2	2	2	2.6	5.14	0.11
393	sp|P32969|R	52	21964	1	1	1	1	9.9	9.96	0.15
394	sp|O43143|D	51	91673	1	1	1	1	1.5	7.12	0.04
395	sp|Q9BZ95|N	51	165330	1	1	1	1	1.6	8.57	0.02
396	sp|Q00534|C	51	37257	2	2	2	2	8.3	6.02	0.19
397	sp|Q02878|R	50	32765	2	2	2	2	6.6	10.59	0.21
398	sp|Q6PJT7|Z	50	83793	1	1	1	1	2.3	6.99	0.04
399	sp|O43592|X	50	111148	1	1	1	1	1.2	5.24	0.03
400	sp|Q96SB3|N	50	89451	1	1	1	1	2.4	4.91	0.04
401	sp|Q9UNX3|	50	17246	2	2	2	2	11	10.55	0.43
402	sp|P28331|N	50	80443	1	1	1	1	2.6	5.89	0.04
403	sp|Q9Y285|S	50	57585	1	1	1	1	2.2	7.31	0.06
404	sp|Q9Y5Y5|P	49	38662	2	2	1	1	6.8	9.85	0.18
405	sp|Q7L9L4|M	49	25246	1	1	1	1	5.1	6.24	0.13
406	sp|P62304|R	49	10854	1	1	1	1	12	9.46	0.32
407	sp|Q9HCE1|I	49	114512	1	1	1	1	1.4	9	0.03
408	sp|O94925|G	48	74269	1	1	1	1	3.1	7.85	0.04
409	sp|P10515|C	48	69466	1	1	1	1	2.3	7.96	0.05
410	sp|O00159|M	48	122461	1	1	1	1	1.5	9.46	0.03
411	sp|Q86Y56|D	48	94774	1	1	1	1	1.3	5.98	0.03
412	sp|Q9NS69|	48	15512	1	1	1	1	7.7	4.27	0.22
413	sp|O94973|A	47	104807	1	1	1	1	1.1	6.53	0.03
414	sp|P61353|R	47	15788	2	2	2	2	24.3	10.56	0.48
415	sp|O14979|H	47	46580	2	2	2	2	4.8	9.59	0.15
416	sp|Q9Y4Z0|L	47	15511	1	1	1	1	11.5	10.02	0.22
417	sp|Q5T2N8|A	47	46635	1	1	1	1	3.4	9.37	0.07
418	sp|Q9UNM6	47	43203	1	1	1	1	3.2	5.53	0.08

419	sp|Q15046|S	46	68461	2	2	2	2	4.2	5.94	0.1
420	sp|P62851|R	46	13791	2	2	2	2	15.2	10.12	0.56
421	sp|Q5JWF2|(	46	111697	1	1	1	1	1.1	4.91	0.03
422	sp|Q16401|P	46	56560	2	2	2	2	5.4	5.35	0.12
423	sp|P53621|C	46	139797	2	2	2	2	1.8	7.7	0.05
424	sp|O15269|S	45	53281	1	1	1	1	3	5.72	0.06
425	sp|P54727|R	45	43202	1	1	1	1	7.1	4.79	0.08
426	sp|Q13884|S	45	58367	1	1	1	1	4.1	8.81	0.06
427	sp|P54819|K	45	26689	1	1	1	1	5.4	7.67	0.13
428	sp|P14406|C	45	9390	1	1	1	1	12	9.75	0.37
429	sp|P46781|R	45	22635	1	1	1	1	4.6	10.66	0.15
430	sp|O43795|N	45	132928	2	2	2	2	1.8	9.43	0.05
431	sp|P47914|R	44	17798	1	1	1	1	9.4	11.66	0.19
432	sp|Q969G9|N	44	52652	1	1	1	1	4	8.82	0.06
433	sp|Q96M96|	44	87598	1	1	1	1	2	5.8	0.04
434	sp|P61970|N	44	14640	2	2	2	2	18.9	5.1	0.52
435	sp|P52788|S	43	41698	1	1	1	1	3	4.87	0.08
436	sp|P54136|S	43	76129	2	2	2	2	3	6.26	0.09
437	sp|Q15417|C	43	36562	2	2	2	2	6.1	5.69	0.19
438	sp|P19474|R	43	55162	1	1	1	1	3.8	5.98	0.06
439	sp|O14787|T	43	102862	1	1	1	1	1.3	4.87	0.03
440	sp|P62280|R	43	18590	2	2	2	2	10.8	10.31	0.4
441	sp|O95831|A	43	67144	1	1	1	1	2.1	9.04	0.05
442	sp|Q14204|D	43	534809	1	1	1	1	0.3	6.01	0.01
443	sp|Q9NZ01|T	42	36410	1	1	1	1	3.2	9.5	0.09
444	sp|O75489|N	42	30337	1	1	1	1	4.9	6.99	0.11
445	sp|P35606|C	41	103278	1	1	1	1	1.4	5.15	0.03
446	sp|P51812|K	41	84025	2	2	2	2	3.9	6.41	0.08
447	sp|Q6DD87|Z	41	41429	1	1	1	1	3.4	8.25	0.08
448	sp|Q9UBB4|A	41	54196	1	1	1	1	2.1	5.12	0.06
449	sp|P08708|R	41	15597	1	1	1	1	19.3	9.85	0.22
450	sp|A0A075B	41	12889	1	1	1	1	7.8	5.13	0.27
451	sp|O15381|N	40	96017	1	1	1	1	1.8	6.11	0.03
452	sp|P43405|K	40	72533	1	1	1	1	1.6	8.43	0.05
453	sp|Q14254|F	40	47434	1	1	1	1	3.3	5.19	0.07
454	sp|P18077|R	40	12587	1	1	1	1	9.1	11.07	0.27
455	sp|Q14571|I	40	311060	1	1	1	1	0.4	6.01	0.01
456	sp|P40429|R	39	23619	2	2	2	2	8.4	10.94	0.3
457	sp|P62701|R	39	29807	1	1	1	1	3.8	10.16	0.11
458	sp|O00231|P	39	47719	2	2	2	2	5.5	6.08	0.14
459	sp|O76041|N	39	116609	1	1	1	1	1.3	7.89	0.03
460	sp|P00505|A	39	47886	1	1	1	1	2.3	9.14	0.07
461	sp|P62244|R	38	14944	1	1	1	1	6.9	10.14	0.23
462	sp|Q9NWS0	38	32513	1	1	1	1	3.4	5.05	0.1
463	sp|Q96HS1|P	38	32213	1	1	1	1	3.5	8.88	0.1
464	sp|Q9Y224|F	37	28165	1	1	1	1	4.1	6.19	0.12
465	sp|O95168|N	37	15256	1	1	1	1	7.8	9.85	0.22
466	sp|Q9NZB2|	37	123008	1	1	1	1	2.2	9.07	0.03
467	sp|Q99623|P	37	33276	1	1	1	1	3.7	9.83	0.1
468	sp|Q9H0E2|T	37	30490	1	1	1	1	5.1	5.68	0.11
469	sp|P41252|S	37	145718	2	2	2	2	1.3	5.82	0.05
470	sp|P62861|R	37	6644	1	1	1	1	16.9	12.15	0.54
471	sp|Q96JI7|SF	37	282681	1	1	1	1	0.4	5.63	0.01
472	sp|P07384|C	36	82465	1	1	1	1	1.1	5.49	0.04
473	sp|P11166|G	36	54391	1	1	1	1	1.6	8.93	0.06
474	sp|Q6PIU2|N	36	46064	1	1	1	1	3.9	6.76	0.07
475	sp|P62841|R	36	17029	2	2	1	1	8.3	10.39	0.44
476	sp|Q00325|N	36	40525	1	1	1	1	2.2	9.45	0.08
477	sp|Q13347|E	36	36878	2	2	1	1	3.4	5.38	0.19
478	sp|A6NHQ2	35	34839	1	1	1	1	3.3	10.35	0.1

479	sp|Q14789|G	35	377215	1	1	1	1	0.3	4.96	0.01
480	sp|P13861|K	35	45832	1	1	1	1	3	4.96	0.07
481	sp|P23526|S	34	48255	1	1	1	1	3	5.92	0.07
482	sp|P01023|A	34	164613	1	1	1	1	0.7	6.03	0.02
483	sp|Q71DI3|H	34	15436	1	1	1	1	8.1	11.27	0.22
484	sp|P78344|I	34	102810	1	1	1	1	1.4	6.7	0.03
485	sp|P09525|A	34	36088	1	1	1	1	5	5.84	0.09
486	sp|P04085|P	33	24598	1	1	1	1	3.8	9.52	0.14
487	sp|P36507|M	33	44681	1	1	1	1	2.8	6.12	0.07
488	sp|Q16822|P	33	71452	1	1	1	1	1.4	7.57	0.05
489	sp|Q8N1G4|	33	64004	1	1	1	1	3.1	8.55	0.05
490	sp|P32119|P	33	22049	1	1	1	1	9.1	5.66	0.15
491	sp|P37837|T	33	37688	1	1	1	1	3.3	6.36	0.09
492	sp|Q6UX68|X	32	75707	1	1	1	1	1.7	6.21	0.04
493	sp|Q06830|P	32	22324	3	3	3	3	14.1	8.27	0.52
494	sp|Q9NQW7	32	70558	1	1	1	1	2.6	5.42	0.05
495	sp|P61313|R	32	24245	1	1	1	1	3.4	11.62	0.14
496	sp|Q9UH17|	32	46749	1	1	1	1	2.9	5.74	0.07
497	sp|O94979|S	32	133900	1	1	1	1	1.5	6.43	0.02
498	sp|Q9C0F3|Z	32	55668	1	1	1	1	1.7	6.75	0.06
499	sp|P45974|U	31	96638	1	1	1	1	1.4	4.91	0.03
500	sp|Q9GZT3|S	31	12398	1	1	1	1	9.2	10.25	0.28
501	sp|P50995|A	31	54697	1	1	1	1	2.2	7.53	0.06
502	sp|P62906|R	30	24987	1	1	1	1	6	9.94	0.13
503	sp|Q9UKF6|	30	78120	1	1	1	1	1.5	5.37	0.04
504	sp|Q9BW04|	30	64211	1	1	1	1	2.8	8.78	0.05
505	sp|O14579|C	29	34688	1	1	1	1	3.6	4.97	0.1
506	sp|Q9NSD9|	29	66701	1	1	1	1	1.5	6.4	0.05
507	sp|O14508|S	29	22443	1	1	1	1	9.1	8.9	0.15
508	sp|Q9H900|Z	29	67685	1	1	1	1	2.2	5.85	0.05
509	sp|O15372|E	29	40076	1	1	1	1	5.1	6.09	0.08
510	sp|Q8WUW1	29	8796	1	1	1	1	14.7	5.35	0.4
511	sp|Q9UQN3	28	23948	1	1	1	1	3.3	8.8	0.14
512	sp|Q9BXJ9|N	28	102462	1	1	1	1	1	7.23	0.03
513	sp|Q9C037|T	28	58280	1	1	1	1	2.2	8.4	0.06
514	sp|P06744|G	28	63335	1	1	1	1	1.3	8.43	0.05
515	sp|Q04828|A	28	37221	1	1	1	1	2.5	8.02	0.09
516	sp|O43809|C	28	26268	1	1	1	1	3.5	8.85	0.13
517	sp|P51946|C	27	38075	1	1	1	1	5	6.73	0.09
518	sp|P10244|M	27	79343	1	1	1	1	1.6	6.43	0.04
519	sp|P23381|SY	27	53474	1	1	1	1	3.4	5.83	0.06
520	sp|Q9P0J0|N	27	16688	1	1	1	1	9.7	8.04	0.2
521	sp|Q9ULE6|F	27	97548	1	1	1	1	1.4	6.08	0.03
522	sp|Q9H3P2|I	27	57469	1	1	1	1	2.7	9.1	0.06
523	sp|Q6NX45|	26	56686	1	1	1	1	2.1	8.71	0.06
524	sp|P42677|R	26	9797	1	1	1	1	13.1	9.57	0.36
525	sp|Q96N67|I	26	244289	1	1	1	1	0.5	6.34	0.01
526	sp|Q08380|L	26	66202	1	1	1	1	2.2	5.13	0.05
527	sp|Q14980|N	26	239199	1	1	1	1	0.6	5.63	0.01
528	sp|P30622|C	26	162888	1	1	1	1	1.6	5.29	0.02
529	sp|O60518|R	26	126173	1	1	1	1	0.6	4.88	0.03
530	sp|Q13501|S	26	48455	1	1	1	1	3.2	5.1	0.07
531	sp|P60981|D	26	18950	1	1	1	1	5.5	8.06	0.18
532	sp|Q13242|S	25	25640	1	1	1	1	4.5	8.74	0.13
533	sp|Q9Y3Y2|C	25	26380	1	1	1	1	6.5	12.24	0.13
534	sp|Q9BX26|S	25	177239	1	1	1	1	0.5	9.01	0.02
535	sp|Q63HK5|T	24	119518	1	1	1	1	1.6	6.83	0.03
536	sp|Q16527|C	24	21852	1	1	1	1	10.9	8.95	0.15
537	sp|P20674|C	24	16923	1	1	1	1	6	6.3	0.2
538	sp|Q9H7E2|T	24	73425	1	1	1	1	1.2	9.27	0.04

539	sp|P60228|E	24	52587	1	1	1	1	2.5	5.71	0.06
540	sp|Q9P2H3|I	24	88664	1	1	1	1	2.4	7.59	0.04
541	sp|Q8NBF2|	23	80249	1	1	1	1	1.2	5.33	0.04
542	sp|P25786|P	21	29822	1	1	1	1	7.6	6.15	0.11
543	sp|Q96LX7|C	21	68078	1	1	1	1	1.6	6.75	0.05
544	sp|Q5S007|L	21	289568	1	1	1	1	0.7	6.35	0.01
545	sp|Q14019|C	21	16049	1	1	1	1	4.2	5.54	0.21
546	sp|Q6PD62|(	21	134332	1	1	1	1	0.5	6.32	0.02
547	sp|Q9C0D6|I	18	125654	1	1	1	1	0.6	9.17	0.03
548	sp|Q9BYJ9|Y	18	61007	1	1	1	1	1.8	8.86	0.05
549	sp|Q86VP6|(	16	137999	1	1	1	1	1	5.52	0.02
550	sp|Q14011|C	15	18637	1	1	1	1	6.4	9.51	0.18
551	sp|Q17RY6|L	14	19288	1	1	1	1	8.5	7.44	0.17
