# Supplementary material for: CircHAS2 activates CCNE2 to promote cell proliferation and sensitizes the response of colorectal cancer to anlotinib
Source: Mol Cancer. 2024 Mar 21;23:59. doi: 10.1186/s12943-024-01971-7 (PMC10956180; doi:10.1186/s12943-024-01971-7)
Supplement: Supplementary file 5 — Supplementary Material 5 [file 12943_2024_1971_MOESM5_ESM.pdf]

Table S4 Baseline clinical characteristics of enrolled patients and best tumor response during treatment

Patient No.	Gender	Age	Lesion & Pathology	TNM stage	Metastasis site	Treatment duration (weeks)	Best response
Group A-1	M	61	Rectum, Adenocarcinoma	T4aN0M1	Liver	21	PR
Group A-2	F	71	Colon, Adenocarcinoma	TxN2aM1	Liver, Peritoneum	18	SD
Group A-3	M	66	Rectum, Adenocarcinoma	TxN1bM1	Liver	24	PR
Group A-4	F	68	Colon, Adenocarcinoma	T4aNxM1	Liver, Lung	21	PR
Group A-5	M	51	Colon, Adenocarcinoma	T4bN1bM1	Liver, Retroperitoneum	24	PR
Group A-6	M	46	Colon, Adenocarcinoma	TxN1bM1	Liver, bone	18	PR
Group A-7	F	35	Colon, Adenocarcinoma	TxN1aM1	Liver	21	PR
Group B-1	M	68	Rectum; Adenocarcinoma	T3N1bM1	Liver	21	SD
Group B-2	M	57	Rectum; Adenocarcinoma	T3N2aM1	Liver	15	PD
Group B-3	F	40	Rectum; Adenocarcinoma	T4aN1bM1	Liver, Retroperitoneum	18	SD
Group B-4	M	64	Colon, Adenocarcinoma	T3N2aM1	Liver, Retroperitoneum	21	PR
Group B-5	M	67	Colon, Adenocarcinoma	T4aN2bM1	Liver	18	SD
Group B-6	F	64	Colon, Adenocarcinoma	T4aN1aM1	Liver	24	PR
Group B-7	M	78	Rectum; Adenocarcinoma	T4aN1bM1	Liver	21	SD

PD, progressive disease; SD, stable disease; PR, partial response.
