# Supplementary material for: CircHAS2 activates CCNE2 to promote cell proliferation and sensitizes the response of colorectal cancer to anlotinib
Source: Mol Cancer. 2024 Mar 21;23:59. doi: 10.1186/s12943-024-01971-7 (PMC10956180; doi:10.1186/s12943-024-01971-7)
Supplement: Supplementary file 6 — Supplementary Material 6 [file 12943_2024_1971_MOESM6_ESM.pdf]

Table S5. Primer sequences, siRNA sequences, and miRNA sequences used in this study

Primers sequences:	
circHAS2 forward	GCTCGCAACACGTAACGCAAT
circHAS2 reverse	GCTGTGATTCCAAGGAGGAGAGA
GAPDH forward	GAGTCAACGGATTTGGTCGT
GAPDH reverse	GACAAGCTTCCCGTTCTCAG
β-actin forward	CAGCCTTCCTTCCTGGGCATG
β-actin reverse	ATTGTGCTGGGTGCCAGGGCAG
U6 forward	CTCGCTTCGGCAGCACA
U6 reverse	AACGCTTCACGAATTTGCGT
miR-1244 forward	AACACGCTTGGTAGAGTATGTTTG
miR-1244 reverse	GTCGTATCCAGTGCAGGGT
CCNE2 forward	AGCTCAGCTTTTAGATCTGTGT
CCNE2 reverse	TTCTGAAATACTGTCCCACTCC
HAS2 forward	AAGAACAACTTCCACGAAAAGG
HAS2 reverse	TACATAATCCACACTTCGTCCC
USP10 forward	AACACGCTTGGTAGAGTATGTTTG
USP10 reverse	GTCGTATCCAGTGCAGGGT
p53 forward	TTCCTGAAAACAACGTTCTGTC
p53 reverse	AACCATTGTTCAATATCGTCCG
p21 forward	GATGGAACTTCGACTTTGTCAC
p21 reverse	GTCCACATGGTCTTCCTCTG
CDK2 forward	CCTGGGCTGCAAATATTATTCC
CDK2 reverse	TGGCTTGTAATCAGGCATAGAA
GAPDH divergent forward	CCCTGTGCTCAACCAGCTCTC
GAPDH divergent reverse	CCGACCTTCACCTTCCCCAT
circHAS2-1 forward	AATTGGAACCACACTCTTTGGAG
circHAS2-1 reverse	AGCCAACAATATAAGCAGCTGTG
circHAS2-2 forward	TTTGGCATCACACCTCATCATCC
circHAS2-2 reverse	GGATCTTCTTGATAGGCAGCGATG
circHAS2-3 forward	AGGCTAACCTACCCTGGGATTAAAG
circHAS2-3 reve rse	ATTTGTCTCTGCCCATGACTTCAC
circHAS2-4 forward	CACGTAACGCAATTGGTCTTGT
circHAS2-4 reverse	CAGTGCTCTGAAGGCTGTGTAC

siRNA sequences	
siRNA-NC	GTTCTCCGAACGTGTCACGTT
si-circHAS2#1	GUGGAUUAUGUACAGAUGCAUTT
si-circHAS2#2	GGAUUAUGUACAGAUGCAUUGTT
si-USP10#1	CACCUGAAACGAUUCGUUUAUTT
si-USP10#2	CGACAAGCUCUUGGAGAUAAATT
si-CCNE2#1	CCCAGATAATCCAGGCCAA
si-CCNE2#2	AGACGAAGTAGCCGTTTAC

miRNA sequences	
miR-1244 mimics sense	AAGUAGUUGGUUUGUAUGAGAUGGUU
miR-1244 mimics anti-sense	AACCAUCUCAUACAAACCAACUACUU
miR-NC sense	CUACAUUAAGCAAAAUCACCATT
miR-NC anti-sense	UACAUUAAGCAAAAUCACCAGTT
miR-1244 inhibitor	AACCAUCUCAUACAAACCAACUACUU
miR-1244 RT	GTCGTATCCAGTGCAGGGTCCGAGGTATTCGCACTG GATACGACTTCATC
