# Supplementary material for: CircHAS2 activates CCNE2 to promote cell proliferation and sensitizes the response of colorectal cancer to anlotinib
Source: Mol Cancer. 2024 Mar 21;23:59. doi: 10.1186/s12943-024-01971-7 (PMC10956180; doi:10.1186/s12943-024-01971-7)
Supplement: Supplementary file 7 — Supplementary Material 7 [file 12943_2024_1971_MOESM7_ESM.pdf]

Table S6. Antibodies used in research

Antibody	Company	Cat. No.	Species	Dilution
GAPDH	Abcolnal	AC002	Mouse	1:1000
β-tubulin	Abcolnal	A12289	Rabbit	1:1000
CCNA2	Cell signaling	4656	Mouse	1:1000
CCNE1	Cell signaling	20808S	Rabbit	1:1000
CCNE2	Cell signaling	4132	Rabbit	1:1000
CCNE2	Abcolnal	A7032	Rabbit	1:1000
CDK2	Cell signaling	18048S	Rabbit	1:1000
CDK2	Abcolnal	A0294	Rabbit	1:1000
CDK1	Cell signaling	9114	Rabbit	1:2000
Histone-H3	Proteintech	17168-1-AP	Rabbit	1:1000
p53	Cell signaling	2524	Mouse	1:2000
p53	Abcolnal	A0263	Rabbit	1:2000
p21	Cell signaling	2946	Rabbit	1:1000
p21	Abcolnal	A19094	Rabbit	1:1000
USP10	Cell signaling	8501	Rabbit	1:1000
USP10	Abcolnal	A13387	Rabbit	1:1000
USP10	Abcolnal	A4454	Rabbit	1:1000
Flag	Abcam	ab205606	Rabbit	1:1000
Flag	Abcolnal	AE092	Rabbit	1:1000
